# Terrestrialization, Miniaturization and Rates of Diversification in African Puddle Frogs (Anura: Phrynobatrachidae)

**DOI:** 10.1371/journal.pone.0035118

**Published:** 2012-04-10

**Authors:** Breda M. Zimkus, Lucinda Lawson, Simon P. Loader, James Hanken

**Affiliations:** 1 Museum of Comparative Zoology and Department of Organismic and Evolutionary Biology, Harvard University, Cambridge, Massachusetts, United States of America; 2 Center for Medical, Agricultural, and Veterinary Entomology, Agricultural Research Services, United States Department of Agriculture, Gainesville, Florida, United States of America; 3 Institute of Biogeography, University of Basel, Basel, Switzerland; Michigan State University, United States of America

## Abstract

Terrestrialization, the evolution of non-aquatic oviposition, and miniaturization, the evolution of tiny adult body size, are recurring trends in amphibian evolution, but the relationships among the traits that characterize these phenomena are not well understood. Furthermore, these traits have been identified as possible “key innovations” that are predicted to increase rates of speciation in those lineages in which they evolve. We examine terrestrialization and miniaturization in sub-Saharan puddle frogs (Phrynobatrachidae) in a phylogenetic context to investigate the relationship between adaptation and diversification through time. We use relative dating techniques to ascertain if character trait shifts are associated with increased diversification rates, and we evaluate the likelihood that a single temporal event can explain the evolution of those traits. Results indicate alternate reproductive modes evolved independently in *Phrynobatrachus* at least seven times, including terrestrial deposition of eggs and terrestrial, non-feeding larvae. These shifts towards alternate reproductive modes are not linked to a common temporal event. Contrary to the “key innovations” hypothesis, clades that exhibit alternate reproductive modes have lower diversification rates than those that deposit eggs aquatically. Adult habitat, pedal webbing and body size have no effect on diversification rates. Though these traits putatively identified as key innovations for *Phrynobatrachus* do not seem to be associated with increased speciation rates, they may still provide opportunities to extend into new niches, thus increasing overall diversity.

## Introduction

Trait shifts that enable extension into and colonization of new environments, or ecospace, and subsequent diversification in ways that were not previously possible are commonly cited as “key innovations” [1], [2]. These characteristics are hypothesized to influence diversification rates, and include traits associated with life history, body size and ecology [3]–[7]. Few studies, however, have analyzed diversification rates within extensively sampled radiations to determine whether specific traits promote speciation due to the difficulty of assembling such comprehensive data sets [8]–[12]. The concept of key innovations is further complicated by the fact that successful adaptation to a new ecological niche must be attributable to a specific innovation. To identify potential key innovations and understand current patterns of species diversity, both intrinsic and extrinsic factors must be investigated simultaneously. Intrinsic characteristics provide the adaptive potential for radiation, while extrinsic factors, such as climate or habitat change, provide the ecological opportunity for diversification.

One possible key innovation in amphibians is the shift to terrestrial reproduction, which allows eggs to be laid away from standing water and facilitates the transition from aquatic to terrestrial existence. Amphibians are unique among tetrapod vertebrates in the tremendous diversity of ways in which they reproduce [13]. The generalized reproductive mode in anurans comprises a biphasic pattern of development, which includes large clutches of small aquatic eggs and free-living, aquatic larvae that undergo metamorphosis to terrestrial adults. There are, however, nearly 40 alternate reproductive modes in anurans, many of which are characterized by small clutches of large terrestrial or arboreal eggs [14]. In some species eggs hatch into non-feeding tadpoles and undergo metamorphosis after utilizing endogenous yolk supplies, whereas others employ direct development and lack the free-living larval stage entirely. This shift to a non-aquatic lifestyle is a recurring theme in amphibian evolution that is manifest as multiple independent occurrences of terrestrialization and direct development [13], [15]–[21]. The shift to an alternate mode of reproduction may enable a lineage to occupy a new ecological niche or habitat, in the same way that loss of the free-living, feeding larval stage may enable populations to persist in areas without permanent bodies of water [13], [22].

Another widespread phenomenon among animals, including amphibians, is miniaturization, the evolution of extremely decreased adult body size. Although many biologists have noted that body size and degree of terrestriality appear to be correlated within certain amphibian lineages, the relationship between these two trends has not been adequately investigated. Body size is undisputedly one of the most significant organismal traits because it influences many biological attributes, including behavior, ecology, life history and physiology. The selective advantages of miniaturization include predator avoidance [23], [24], exploitation of alternate food sources [23], utilization of physically smaller niches [23], [25], [26] and attainment of reproductive maturity at an earlier age [24], [27]. Miniaturization likely played an important role in the origin of the Lissamphibia, which includes all living amphibians, and is a recurring trend within all three extant orders. Miniaturization has been documented within anurans, caecilians and salamanders and has occurred independently many times in each clade [13], [23], [28]–[33]. Although miniaturization is an important factor in amphibian evolution, its relation to species diversification and its correlation with other proposed key innovations are poorly understood; recent studies have yielded conflicting results. In the global radiation of toads, the most speciose clades were characterized by large body sizes and large ranges [34]. In contrast, the most speciose clades of Malagasy mantellid frogs were characterized by small body size and small ranges, which was attributed to the poor dispersal ability of small-bodied species [35]. Finally, the diversity of Amazonian hylid treefrogs is not explained by body size evolution, but rather by the timing of colonization and long-term sympatry of multiple clades [36].

Puddle frogs of the genus *Phrynobatrachus* (Anura: Phrynobatrachidae) comprise approximately 80 species distributed across sub-Saharan Africa, where they are adapted to aquatic, semi-aquatic, and terrestrial habitats [37]–[39]. They comprise the second largest generic radiation on the continent, which makes them ideal for investigating broader evolutionary studies of continental speciation. Adult snout-vent lengths (SVL) vary greatly, from as little as 12 mm in some miniaturized species to greater than 50 mm in the largest species. The lineage also displays diverse reproductive modes. Although most species deposit large clutches of aquatic eggs, some species are semi-independent from permanent water and lay eggs in very small amounts of water where the tadpoles develop and feed [40]–[44]. Other species are endotrophic, with terrestrial eggs and non-feeding larvae that obtain developmental nutrients entirely from maternally provisioned yolk.

Recent derivation of a molecular phylogeny of sub-Saharan puddle frogs enables us to simultaneously investigate terrestrialization and miniaturization for the first time [39]. Here we use this phylogeny, reanalyzed with improved species-tree methods, to test the following hypotheses: (1) terrestrialization is characterized by alternate modes of reproduction and low water dependence due to occupation of habitats with high humidity; (2) alternate reproductive modes evolved once within puddle frogs; (3) miniaturization is correlated with decreased pedal webbing; and (4) miniaturization and decreased pedal webbing are correlated with terrestrialization.We also use relative dating methods and diversification analysis to test two additional hypotheses: (5) character traits (e.g., reproductive mode, adult habitat, pedal webbing and body size) are significantly correlated with change in diversification rates within *Phrynobatrachus*, and (6) a single temporal or geographic events correspond to shifts in character trait changes across the *Phrynobatrachus* phylogeny, thereby implying a common extrinsic cause. Testing these hypotheses allows us to determine if morphological (intrinsic) and ecological (extrinsic) traits that characterize terrestrialization impact diversification patterns within puddle frogs, and possibly all amphibians, thereby allowing them to take advantage of resources not previously utilized by aquatic clades.

## Methods

### Taxon sampling and specimen preparation

All field collections made for this study were obtained with appropriate permits (Ethiopia: Wildlife Conservation Department collection permit 13/37/55/521, export permit 13/37/57/568; Tanzania: COSTECH research permit 2007-45-NA-2007-3, Trophy Export Certificate No. 50086-89). External morphology was assessed by examination of alcohol-preserved specimens (Table S1). A total of 279 *Phrynobatrachu*s specimens were examined, comprising 54 species, as well as nine outgroup genera. Only adults were included; mature specimens were identified by the presence of secondary sexual characteristics and/or ossified carpal bones (using radiography). Radiographs were obtained with a Thermo Kevex digital x-ray (Model PXS10) in combination with a PaxScan amorphous silicon sensor array (Model 4030R) and ViVa version 2.0 (Varian Medical Systems, Inc.); specimens were x-rayed at 40 kV.

### Phylogenetic relationships and relative dating

Previous hypotheses of *Phrynobatrachus* phylogeny were obtained by using maximum parsimony (MP), maximum likelihood (ML) and Bayesian (BI) methods for three datasets: mitochondrial DNA (mtDNA; approximately 2.5 kilobases, including 12S rRNA, valine-tRNA and 16S rRNA), nuclear gene RAG-1 (920 base pairs; bp) and combined partitions (mtDNA+RAG-1) [39]. For this study, the combined-dataset phylogeny of Zimkus et al. (2010) [39] was reanalyzed with improved species-tree methods implemented using the program *BEAST (starBEAST; BEAST package version 1.6.1) [45]. The consensus nuclear allele (after all SNPs within individuals were coded as missing information) for RAG-1 was matched to the mtDNA alignment for each specimen to create a 1∶1 mtDNA∶nuDNA dataset. The RAG-1 locus was analyzed with the codon-based SRD06 nucleotide substitution model [46], whereas mtDNA was run using the model selected as most closely fitting the data through AIC, AICc and BIC scores with jModelTest 0.1.1 [47], [48]. jModelTest performs poorly when there is a large amount of missing data. The dataset therefore was trimmed to exclude sites with a high proportion of gaps, which yielded a reduced dataset of 2318 bp, and then further reduced to include only individuals with greater than 90% completeness of the chosen loci, which yielded 165 individuals. Likelihood scores were produced with the full set of possible models (88) using an estimated BIONJ tree topology. The model with the highest likelihood supported by BEAST was GTR+I+G. Substitution-model parameters were unlinked across data partitions, with scaled mutation rates (1∶15 nuDNA∶mtDNA) and strict molecular clocks. Strict clocks were used as relaxed clocks did not significantly improve the analysis. This scaled mutation rate reflects estimates from Crawford (2005) [49] and Lawson (2010) [50], who found a 10–20-fold increase in mutation rate for mtDNA. The chain was 25 million generations, logging every thousandth. The first 5.5 million trees were discarded as burn-in in accordance with a significant and sustained improvement in likelihood after this point (as assessed in TRACER) [51]. Quality of the run was assessed in TRACER; each run was deemed suitable if the posterior had an ESS score above 200 with good mixing in the trace. The tree was run first with an unconstrained topology to determine clades in an unrooted system, and then was run constrained with the basal relationship of *Phrynobatrachus* clade A [39] to root the *BEAST maximum credibility tree. See supplementary materials for *BEAST XML file (Appendix S1).

Specimens identified as *Phrynobatrachus mababiensis* A, B and C are each a distinct species [39]; specimens of *P. mababiensis* B were unavailable for morphological examination, so this species was excluded from character analyses. Provisional identification of three specimens as *Phrynobatrachus mababiensis* A is based solely on geography; these specimens were collected closer to the locality of the specimen used to define this clade (MV 10.8; Namibia: Rundu) [39] than to the localities of *P. mababiensis* B and C. *Phrynobatrachus natalensis* was treated as a single species, although it likely comprises a complex of several species in a monophyletic group [39], [52]. Species identifications follow Zimkus et al. (2010) [39] for the following putative new species: *P.* aff. *latifrons; P. calcaratus* A and B; *P. werneri* A and B; *P.* cf. *hylaois*.

Increased terrestriality is associated with species that exhibit alternate reproductive modes, including terrestrial deposition of eggs and/or tadpoles; see (c) *Character coding and ancestral state reconstruction*. To evaluate hypotheses of temporal congruence of terrestriality, we tested the null hypothesis that contemporaneous divergences explain the origins of species with alternate reproductive modes. Molecular dating techniques require calibration points but, as with many amphibian lineages, fossils are not available for the Phrynobatrachidae or any related African ranoids. In addition, geological vicariance-based events are not ideal calibration points because their use assumes that a specific event led to vicariant speciation [53], [54]. Therefore, we inferred relative rather than absolute time scales from molecular data, which is a method recently used by Loader et al. (2007) [55] to determine if disjunct distributions of African caecilians are temporally congruent. We fixed the root node of the *BEAST consensus tree to an arbitrary value (10) and inferred the relative divergence times of those species that exhibit alternate reproductive modes. Confidence intervals (95%) for estimated divergence dates were used to accept or reject the hypothesis that species with increased reproductive terrestriality are temporally congruent. Regional temporal congruence was also evaluated by coding species according to distribution within five geographic zones based on biotic regions [56], which were used by Zimkus et al. (2010) [39] to reconstruct the historical biogeography of Phrynobatrachidae. These zones are (1) western Africa (west of, but not including, Nigeria; includes the Upper Guinean Forest Zone); (2) central Africa (including the Lower Guinean Forest Zone, Cameroon Volcanic Line, and lowland forests of the Congo River Basin); (3) Albertine Rift Mountains (including eastern Democratic Republic of Congo, Burundi, Rwanda and Uganda); (4) eastern Africa (including the Eastern Arc Mountains); (5) southern Africa (countries south of Malawi and excluding northern Mozambique). Species that are distributed in more than one geographic zone are coded according to the ancestral state of its most recent common ancestor [39].

### Character coding and ancestral state reconstruction

We categorically coded and reconstructed the evolution of four traits: two associated with terrestrialization (reproductive mode and adult habitat) and two associated with miniaturization (pedal webbing and body size). All discrete and multi-state characters were reconstructed using maximum likelihood (ML) in Mesquite on a set of 27,000 post burn-in trees obtained from *BEAST analyses. These analyses were completed under a single-rate Mk likelihood model (mk1) with the likelihood decision threshold set to 1.0 (default). Adult body size and extent of pedal webbing were coded as continuous characters and reconstructed on a pruned consensus tree of the combined dataset using weighted squared-change parsimony in Mesquite 2.01 [57]. Those species for which body size data are not available were excluded from all character evolution analyses and ancestral state reconstructions. We also conducted tests of phylogenetic signal using Pagel's λ [58] calculated in R v. 2.11.1 [59]; a λ value of 0 implies evolution independent of the phylogenetic tree, whereas a value of 1 indicates that the probability of shared inheritance between species is proportional to their relatedness. If a trait exhibits phylogenetic signal, we expect that the phylogeny would be useful in explaining the distribution of character values, but if the phylogenetic signal is low, our ability to infer patterns of character evolution are limited.

#### Reproductive mode

Data regarding reproductive mode were gathered from both published literature and the IUCN Global Amphibian Assessment (Table S2) [60]. Reproductive mode was coded as a binary character for diversification rates analyses: (0) aquatic eggs with feeding, aquatic tadpoles, or (1) alternate reproductive mode. Reproductive mode was also coded as a multi-state character for character evolution analyses: (0) aquatic eggs with feeding, aquatic tadpoles; (1) terrestrial eggs with aquatic tadpoles; (2) terrestrial eggs with terrestrial tadpoles; or (3) direct development, metamorphosed froglets hatch directly from terrestrial eggs. Species without documented reproductive mode data were assumed to have aquatic eggs and larvae. Females of those species were dissected to determine egg size whenever possible. Presence of large eggs was considered indicative of terrestrial clutches, but this condition was not found in any specimen examined.

#### Adult habitat: independence from water bodies

Adult habitat was divided into two categories by reorganizing the IUCN habitats classification scheme [60]. Species were coded as (0) dependent on temporary or permanent water bodies, or (1) semi-independent or independent from temporary or permanent water bodies. We assumed low water dependence if the preferred habitat included only areas of high humidity or damp substrates in one or both of these categories: subtropical/tropical moist lowland forest (IUCN code: 1.6) or moist montane forest (IUCN code: 1.9).

#### Pedal webbing

Extent of pedal webbing is variable among *Phrynobatrachus* species; it ranges from (0) absent or rudimentary with 3.0–4.0 phalanges free on toe IV, to (1) moderate to extensive with 0–2.9 phalanges free on toe IV. In addition to this binary classification, we created both continuous and categorical datasets of pedal webbing. The continuous measure was calculated by quantifying the number of phalanges free on the fourth toe (Table S1); the maximum value quantified was used as an heuristic measure for the species. If the extent of webbing attached to one side of the fourth toe differed from that on the other side, the foot was classified with more extensive webbing and fewer phalanges free. Evolution of pedal webbing was also reconstructed as a discrete character by allocating data among five categories: (1) webbing absent, 4 phalanges free on toe IV; (2) rudimentary webbing, 3–3.9 phalanges free on toe IV; (3) moderate webbing, 2–2.9 phalanges free on toe IV; (4) moderate webbing, 1–1.9 phalanges free on toe IV; and (5) extensive webbing, 0–0.9 phalanges free on toe IV.

#### Body size

Evolution of body size was investigated by using maximum values for each species. Snout-vent length (SVL) was determined from direct measurements of alcohol-preserved specimens using digital calipers (±0.1 mm; Table S1); all measurements were taken by one person (BZ). Puddle frog females are often larger than males, but there are many exceptions. In addition, species either lack external morphological characters that reliably distinguish between sexes, or male secondary sexual characteristics may be exhibited only during the breeding season [38]. Therefore, maximum body size for each ingroup species was estimated by using the largest SVL measured, regardless of sex. Maximum SVL approximates the upper bounds for each species; its use enables the inclusion of specimens of both sexes. These values serve as an heuristic measure of maximum body size because of error associated with different sampling intensities among species [33]. Data from the literature were utilized to determine maximum body size for a particular species if few or no specimens were available for examination. Maximum SVL for outgroup genera was estimated by using data from the literature (Table S1). Previous studies of anuran body size evolution utilize binary characters to examine reduction in size and suggest that species smaller than 20 or 25 mm SVL should be considered miniature [23], [32]. This method is not optimal because it designates an arbitrary threshold to distinguish miniaturized versus non-miniaturized taxa. We therefore coded and reconstructed adult body size as binary, multistate and continuous characters to maximize both refinement and power in analyses. We used results of ancestral reconstructions of the continuous characters to investigate possible thresholds for multistate and binary analyses. As a binary character, body size was coded as (0) small, SVL 10–25 mm, or (1) large, SVL≥25.01 mm; as a multistate character, body size was coded as (1) 10–17.5 mm; (2) 17.51–25 mm; (3) 25.01–40 mm; (4) 40.01–55 mm; and (5) ≥55.01 mm.

### Character correlation

We used a continuous-time Markov model implemented in the Discrete module of BayesTraits [61]–[63] to investigate relationships between specific pairs of binary traits: (1) reproductive mode and habitat; (2) reproductive mode and maximum webbing; (3) reproductive mode and snout-vent length; (4) maximum pedal webbing and habitat; (5) maximum pedal webbing and snout-vent length; (6) snout-vent length and habitat. BayesTraits takes into account phylogenetic relationships among species for the across-species analysis of comparative data. To incorporate phylogenetic uncertainty, we used 27,000 post burn-in trees obtained generated from *BEAST analyses. Evidence for correlated evolution is obtained when the dependent model (H_0_) fits the data better than the independent model (H_1_). The likelihood ratio (LR) statistic compares the goodness-of-fit and is defined as follows: LR = 2 log [log-likelihood (H_0_)−log-likelihood (H_1_)]. The LR is then compared to χ^2^ with d.f. = 1 (χ^2^ = 3.84 with a confidence threshold of 0.05).

### Diversification rates

To assess the effects of reproductive mode, adult habitat preference, pedal webbing and body size on diversification of puddle frogs, we completed diversification analyses using a species tree generated from *BEAST. Differential diversification was tested using BiSSE [64] as implemented through the Diversitree package in R v. 2.11.1 [59], [64] using discrete binary characters (previously outlined). We estimated the parameters λ0, λ1, μ0, μ1, q01 and q10 for the species-tree, allowing each binary character state to have unique diversification, extinction and transitions. BiSSE analyses were run with the correction for incomplete sampling unbiased for character state following FitzJohn et al. (2009) [65]. Of the 82 recognized species of *Phrynobatrachus* [37], 58 were used in these analyses. We then ran a Likelihood Ratio Test (LRT) that compared this tree to a tree constrained to have equal diversification rates regardless of character state to determine if the character-dependent speciation model was a significant improvement over a character-independent model (for additional details regarding these analyses, see tutorial at http://www.zoology.ubc.ca/prog/diversitree/).

## Results

### Phylogenetic species-tree relationships

**Figure 1:**
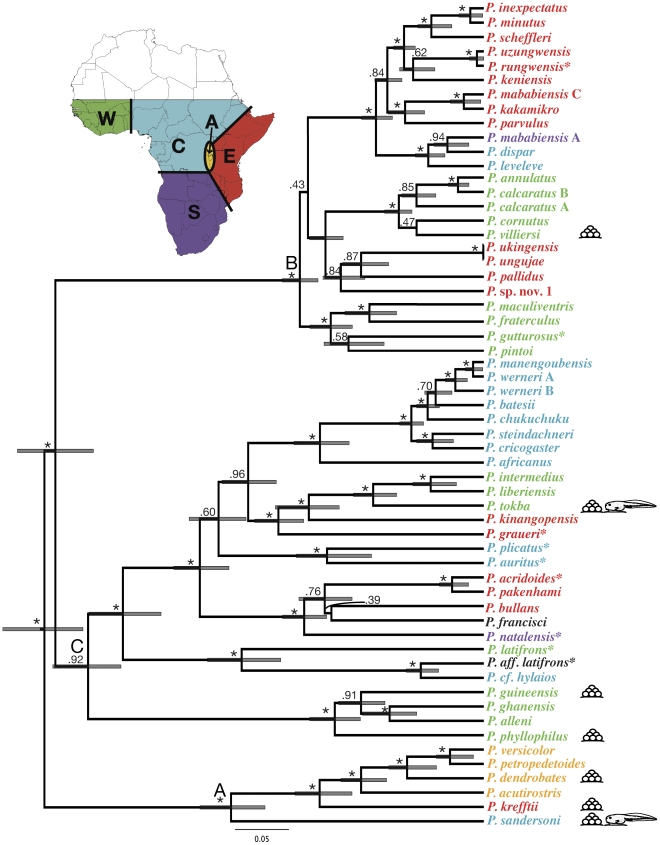
Chronogram of puddle frogs diversification and origins of terrestrialization showing relative time reconstructed using *BEAST. Species are color-coded according to their distribution within five geographic zones (see map inset): (1) western Africa, green; (2) central Africa, blue; (3) Albertine Rift, orange; (4) eastern Africa, red; and (5) southern Africa, purple. Each species marked with an asterisk (*) is present in two biogeographic zones and was color-coded according to the ancestral state of its most recent common ancestor [39]. Species or genera coded in black are present in more than one zone and cannot be assigned unambiguously to a zone by ancestral state. Species that deposit terrestrial eggs or have terrestrial tadpoles are indicated by the egg and tadpole images on the right. Numbers above branches are posterior probabilities; asterisks (*) indicate values greater than 0.95. The three major clades of puddle frogs are indicated (A–C).

Phylogenetic reconstructions using *BEAST yielded the same three major clades identified by Zimkus et al. (2010; Fig. 1, A–C) [39]. All relationships within clade A are both well supported and concurrent with recent phylogenetic reconstructions [39]. Similarly, most nodes are well supported within clade C, which differs only slightly from Zimkus et al. (2010) [39]. Clade B shows somewhat different relationships from Zimkus et al. (2010) [39] with the ancestor of the group likely originating in West Africa instead of East Africa (Fig. 1). Several nodes within clade B have lower bootstrap support values due to missing data in the corresponding taxa. Additional sequence data may be required to resolve relationships within this group.

### Evolution of reproductive mode and independence from water

**Figure 2:**
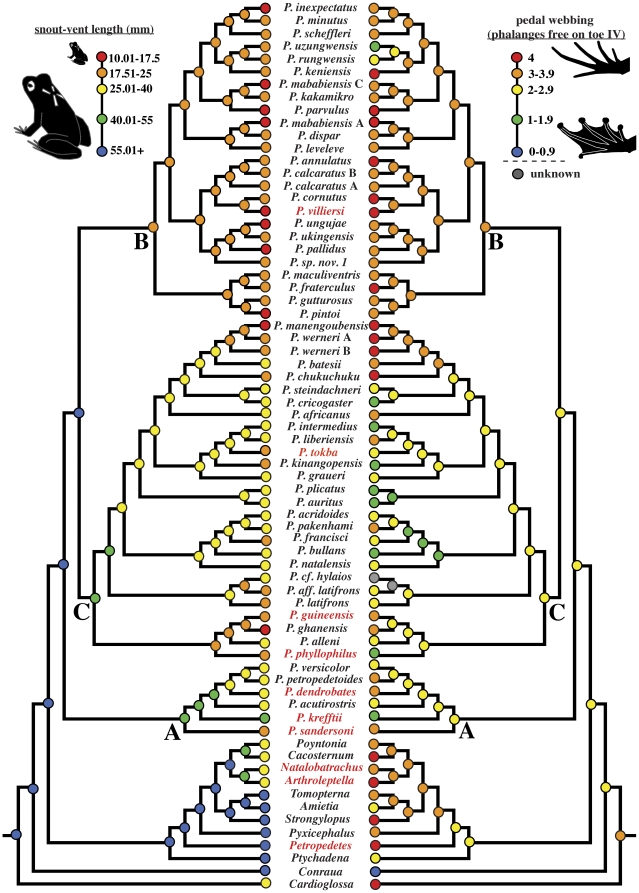
Evolution of body size and pedal webbing in *Phrynobatrachus*. Ancestral state reconstructions for body size (maximum adult snout-vent length) and webbing (minimum number of phalanges free on toe IV for a given species) are mapped to each node using squared-change parsimony. Continuous snout-vent length values have been allocated among five bins: (1) 10.01–17.5 mm (red); (2) 17.51–25 mm (orange); (3) 25.01–40 mm (yellow); (4) 40.01–55 mm (green); (5) >55.01 mm (blue). Numbers of phalanges free of webbing on toe IV have been allocated among five bins: (1) webbing absent, 4.0 phalanges free (red); (2) webbing rudimentary, 3–3.9 phalanges free (orange); (3) webbing moderate, 2–2.9 phalanges free (yellow); (4) webbing moderate, 1–1.9 phalanges free (green); (5) webbing extensive, 0–0.9 phalanges free (blue). Gray circles denote either missing data or those internal nodes for which reconstructions are not possible. Species with alternate reproductive modes are shown in red. The three major clades of puddle frogs (A–C) are indicated.

Most *Phrynobatrachus* species breed in small, lotic bodies of water and have aquatic eggs with free-living, feeding tadpoles. Yet, reproductive modes that provide autonomy from permanent water bodies have evolved independently at least seven times (Figs. 1, 2). Both medium-sized and miniaturized taxa appear to exhibit alternate reproductive modes (Table S2). Only seven species of *Phrynobatrachus* are semi-independent or independent from temporary or permanent water bodies; all inhabit subtropical/tropical moist lowland forest and/or moist montane forest. Each species with low water dependence also exhibits an alternate reproductive mode (Figs. 1, 2). Reproductive mode and adult habitat did not show a strong phylogenetic signal (both: p = 1, λ<0.001) using Pagel's λ [58], thus ancestral character states were not reconstructed.

### Evolution of pedal webbing and body size

Pedal webbing and body size both showed a strong phylogenetic signal (webbing: p = 0.0005, λ = 1; body size: p<0.0001, λ = 1) using Pagel's λ [58]; therefore, ancestral states could be inferred for these two traits. Ancestral state reconstructions reveal a trend towards reduction of pedal webbing within each major clade of puddle frogs. The basal-most clade (Fig. 2, A; Fig. S1) includes species with moderate-to-extensive webbing (1–3.9 phalanges free of webbing on toe IV). The second major clade (B) includes species with absent, rudimentary or moderate webbing (2–4 phalanges free). No species within this clade exhibits extensively or completely webbed toes, and only one species, *P. uzungwensis*, exhibits moderate webbing (1–1.9 phalanges free). The third major clade (C) exhibits extensive variation among species, including absent, rudimentary, moderate and extensive webbing. The most recent common ancestor (*mrca*) of the puddle frog lineage is reconstructed as having moderate webbing (2–2.9 phalanges free).

Ancestral state reconstructions of adult body size reveal patterns similar to those for pedal webbing within each of the three major clades. The basal-most clade (Fig. 2, A; Fig. S2) includes most species with mean SVL greater than 25 mm and is the only clade of puddle frogs with species that exceed 40 mm. Evolution of extremely small body size (mean SVL less than 17.5 mm) has occurred ten times within the two remaining major clades (B, C). All species within the second major clade (B) have mean SVL less than 25 mm, and several of these species are smaller than 17.5 mm. The last major clade (C) also exhibits repeated reduction of body size, with seven independent instances of reduction from an ancestral SVL between 25 and 39.9 mm to less than 25 mm. The reconstructed SVL of the *mrca* of the phrynobatrachid lineage—58 mm—is significantly larger than most extant species.

### Comparative analyses and diversification rates

**Figure 3:**
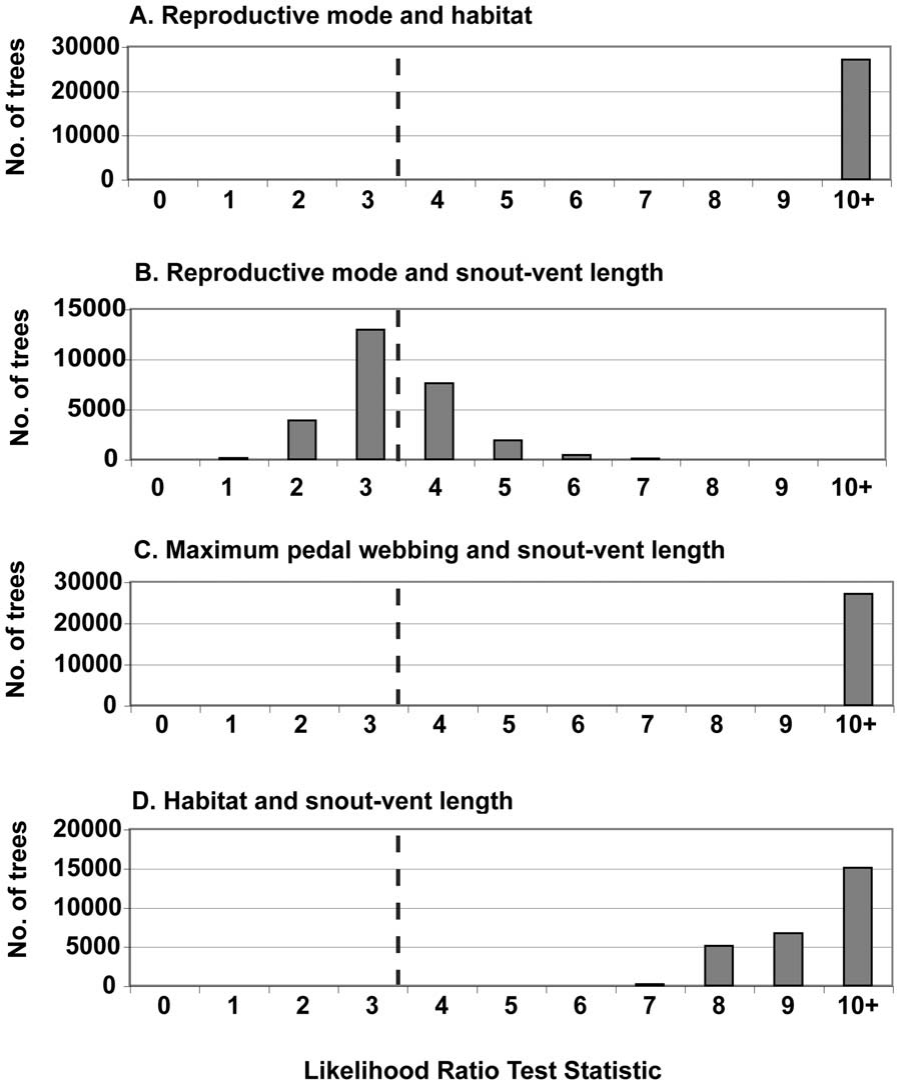
Results of selected correlation tests between pairs of binary characters using BayesTraits. Frequency distributions of likelihood-ratio values were estimated from 27,000 trees (post burn-in trees obtained from *BEAST). Dashed lines indicate significant *p*-values at the 5% threshold. (a) Reproductive mode and adult habitat; (b) reproductive mode and snout-vent length; (c) maximum pedal webbing and snout-vent length; (d) adult habitat and snout-vent length.

Reproductive mode is highly correlated with adult habitat (Fig. 3A), but there is no correlation with pedal webbing. Although likelihood-ratio test statistics for reproductive mode and snout-vent length are not significant, some correlation is apparent (Fig. 3B). Likelihood-ratio tests indicate that body size is correlated with pedal webbing and adult habitat (Fig. 3C, D), yet adult habitat is not correlated with pedal webbing. In BiSSE analyses corrected for random incomplete sampling, only reproductive mode is associated with a change in diversification rate (LRT, p = 0.0487; Table 1). Though independence from temporary or permanent water bodies, pedal webbing and body size were non-significant in relation to changes in diversification rates (p = 0.0592, p = 0.0741, and p = 0.2160, respectively; Table 1), there is a clear relationship between characteristics associated with terrestriality and decreased diversification rate. Extinction rates were consistently not impacted by character state.

**Table 1:** Results of diversification analyses using the species tree generated from *BEAST and the discrete binary characters outlined below in BiSSE as implemented through Diversitree.

		λ0	λ1	μ0	μ1	q01	q10	lnL	deltaAIC	χ^2^	p-value
Reproductive mode	λ0≠1	1.01E+01	3.92E−07	5.56E−07	6.17E+00	1.87E+00	1.58E−04	39.959	-	-	-
	λ0 = 1	1.01E+01	-	2.48E−07	1.94E+01	2.56E+00	1.95E−07	38.016	1.886	3.89	0.0487*
Adult habitat	λ0≠1	1.01E+01	1.01E−05	8.86E−07	6.69E+00	1.92E+00	1.39E−06	39.037	-	-	-
	λ0 = 1	9.51E+00	-	7.61E−10	5.59E+00	2.22E+00	8.74E+00	37.257	1.560	3.57	0.0592
Pedal webbing	λ0≠1	1.16E+01	6.91E+00	1.49E−09	7.53E−08	1.27E+00	2.74E+00	30.115	-	-	-
	λ0 = 1	9.83E+00	-	4.51E−10	2.48E+00	1.42E+00	2.60E+00	28.520	1.189	3.19	0.0741
Body size	λ0≠1	1.09E+01	7.78E+00	1.55E−06	5.54E−07	4.465E−01	2.54E+00	33.489	-	-	-
	λ0 = 1	9.28E+00	-	7.19E−09	5.70E−01	4.43E−01	2.59E+00	32.724	0.469	1.53	0.2160

### Temporal Congruence

We reject the null hypothesis of temporal congruence with a single, common biogeographic event that explains the terrestrialization of individual *Phrynobatrachus* species, using the confidence intervals to discriminate between hypotheses (Fig. 1). Regional temporal congruence also is rejected: many species within the same or adjacent geographic zones that exhibit alternate reproductive modes did not diversify contemporaneously (Fig. 1). Species within adjacent geographic zones that exhibit terrestrialization also do not exhibit temporal congruence; *P. dendrobates* (Albertine) and *P. krefftii* (East) have non-overlapping confidence intervals, and *P. sandersoni* (Central) and the four species from West Africa that exhibit alternate reproductive modes also have non-overlapping confidence intervals. Confidence intervals of four of the six West African pairs do overlap (*P. guineensis* and *P. tokba*; *P. phyllophilus* and *P. tokba*; *P. guineensis* and *P. villersi*; *P. tokba* and *P. villersi*).

## Discussion

Key innovations can confer the adaptive potential required to utilize a resource in a way not previously possible, and such traits may be correlated with subsequent species diversification if radiations into new environmental niches are successful. The transition from water to land is one of the major evolutionary milestones in the history of life, which typically requires a number of significant changes to both morphology and ecology. We thus hypothesized that traits characterizing terrestrialization in puddle frogs might represent key innovations, which enable colonization of environments that are uninhabited by aquatic species. Indeed, puddle frogs have repeatedly become independent from aquatic habitats, with alternate reproductive modes and low water dependence evolving multiple times independently. Yet, and contrary to our hypothesis, puddle frog clades with alternate reproductive modes and low water dependence actually have lower rates of diversification than clades that exhibit biphasic life histories with aquatic egg deposition and high water dependence. Though the traits that characterize terrestrialization enabled species to survive without permanent sources of water, complete terrestriality did not lead to increased diversification. Therefore, we conclude that even though an intrinsic change may have provided the adaptive potential for radiation, without an increase in moist terrestrial climates to facilitate this lifestyle, terrestriality did not increase the ecological opportunity for diversification in the *Phrynobatrachus*.

### Terrestrialization

Most puddle frog species exhibit aquatic egg development, but terrestrial reproductive modes appears to have evolved in *Phrynobatrachus* at least seven times. This recurring trend towards terrestrialization is intriguing: although it may have allowed colonization of new niches, it has neither maintained nor elevated diversification rates. Instead, diversification rates decrease significantly in clades that evolve traits associated with terrestrialization. While this is a significant trend in *Phrynobatrachus*, a correlation between terrestrial development and decreased diversification remains to be investigated in anurans more generally. In addition, plasticity of reproductive mode is understood only superficially in anurans [66]. In addition to confirming the reproductive mode(s) of species for which we hypothesized aquatic reproduction, future investigations of terrestrialization in *Phrynobatrachus* should also consider abiotic factors, such as light level, temperature, and humidity, to determine the degree of plasticity of reproductive mode (i.e., aquatic and/or terrestrial egg deposition) within individual species. Although reproductive plasticity, if found in *Phrynobatrachus*, might obscure the adaptive significance of terrestrialized or aquatic strategies, it would provide a more complete understanding of the shift from aquatic to terrestrial development.

### Miniaturization

We hypothesized that miniaturization is correlated with terrestrialization, but our analysis reveals that these two trends generally are decoupled, even though each has evolved multiple times. Miniaturized body size and reduction of pedal webbing recur within each major clade of puddle frogs. Clade A, which includes large species with moderate-to-extensive webbing, displays only slight reduction in either body size or webbing relative to its most recent common ancestor. The second major clade (B) includes only small species in which webbing generally is rudimentary or absent. The smallest species within clade B are neither one another's closest relatives nor do they occupy the same geographic region. Instead, miniaturized body size evolved at least eight times independently within this group. Clade C also exhibits recurrent body size reduction. However, body sizes in this clade are generally larger than those in clade B, and there are rare instances of body size increase. Webbing is highly variable within clade C, ranging from absent or rudimentary in some species to extensive in others, but such differences are highly correlated with body size. Neither pedal webbing nor body size was correlated with diversification rate.

Reduction of pedal webbing in puddle frogs may be a direct consequence of miniaturization. It is well known that miniaturized amphibians reduce or even lose components of their limbs, including bones of the hands and feet and even whole digits [32], [67]–[69]. The correlation between body size and pedal webbing in puddle frogs also is not surprising given that most small species inhabit leaf-litter as adults (the largest species are semi-aquatic). The link between miniaturization and exploitation of leaf-litter and moss habitats has been discussed in relation to many other frog groups [70]–[73]. Similar correlations between body size and ecology are evident in other amphibians. For example, the largest salamander species are fully aquatic, whereas the smallest species are terrestrial or arboreal [74]. It is somewhat surprising, however, that pedal webbing is not associated with either reproductive mode or water dependence. Although we hypothesized that reduction of pedal webbing would be advantageous to species that are independent of aquatic environments, it is possible that webbing confers an advantage, or at least does not confer a disadvantage, in terrestrial environments. Foot webbing can help maximize forward thrust during swimming, but it also may greatly increase the surface area of the foot that comes in contact with terrestrial surfaces, such as rocks or leaves, thereby conferring increased stability. Future studies should assess the degree of terrestriality of species within this lineage by quantifying time within aquatic environments to more fully resolve the correlation between ecology and pedal webbing.

Small body size is correlated with increased species diversity in numerous lineages [3], [75], [76]. Although body size is not significantly correlated with diversification rate, it is highly probable that size reduction was a key factor in the successful radiation of puddle frogs across sub-Saharan Africa. Size decrease may have enabled smaller species to populate new microhabitats or niches, allowing them to coexist with larger puddle frog species. Whereas the overall trend of decreasing body size may have facilitated the successful radiation of this group, extreme miniaturization does not appear to lead to increased diversification. Interestingly, both miniaturization and direct development are hypothesized to be key traits that allowed the successful range expansion of African squeaker frogs (*Arthroleptis*) from Central Africa into adjacent biogeographic regions [33]. However, the appearance of these characteristics at the base of *Arthroleptis* phylogeny and the predominant trend of increasing body size in that genus contrasts with the patterns of repeated terrestrialization and decreasing body size observed in *Phrynobatrachus*.

### Temporal and spatial patterns of terrestrialization

The extensive geographic range, substantial intrapopulational variation and tiny adult body size of many species of *Phrynobatrachus* are some of the reasons that the systematics of this genus has until recently been poorly understood. However, our understanding of puddle frog diversity, like that of many other tiny vertebrates, has been greatly improved with the advent of molecular approaches. Recent identification of a large number of undescribed and often cryptic species, many of which are extremely small, and the construction of a robust molecular phylogeny allow us to begin to unravel the complex patterns of speciation within this lineage [39], [52], [77]–[79]. Though the current dataset and recently published phylogeny [39] provide much more clarity than ever before, greater geographical sampling and the inclusion of additional loci in future phylogenetic analyses would strengthen our understanding of relationships within a lineage that exhibits extreme diversity in both size and degree of terrestriality.

Terrestrial breeding modes have evolved repeatedly within *Phrynobatrachus* and are not linked to a single biogeographic event or recent time period. This complex evolutionary history contrasts with that of most of Africa's fauna and flora, which are significantly impacted by frequent, recent, continent- and regional-scale drying events [80], [81]. For amphibians with restrictive habitat requirements (e.g., generally moist habitats), these fluctuations would have led species to adapt to dry habitats at continental or regional scales. This pattern was recently documented for the global radiation of toads, but speciation was scaled over a longer period and was not confined to recent Pleistocene changes [34]. Puddle frogs exhibit a complex pattern characterized by multiple, asynchronous events that explain the origins of terrestrialized breeding strategies. Although a single temporal event cannot explain the Africa-wide terrestrialization of puddle frogs, temporal congruence of speciation of some West African species exhibiting alternate reproductive modes suggests that they may have become terrestrialized in response to a common local cause. Given the decrease in diversification rate that accompanies changes in reproductive mode, we conclude that evolution of terrestrialization is not a precursor to radiation in puddle frogs. It does, however, confer the adaptive potential for some species to successfully inhabit and diversify in areas without permanent water.

## Supporting Information

Figure S1 **Evolution of pedal webbing.** Ancestral state reconstructions of the extent of webbing (minimum number of phalanges free on toe IV) are mapped on each node using using maximum likelihood (ML) in Mesquite on a set of 27,000 post burn-in trees. Number of phalanges free of webbing on toe IV are allocated among five bins: (1) webbing absent, 4 phalanges free (white); (2) webbing rudimentary, 3–3.9 phalanges free (blue); (3) webbing moderate, 2–2.9 phalanges free (green); (4) webbing moderate, 1–1.9 phalanges free (yellow); (5) webbing extensive, 0–0.9 phalanges free (black). Red indicates the fraction of trees for which that node is not present; nodes with equivocal reconstructions are indicated in grey. The three major clades of puddle frogs (A–C) are indicated.
(TIF)

Figure S2 **Ancestral state reconstructions for body size (maximum adult snout-vent length) mapped on each node using maximum likelihood (ML) in Mesquite on a set of 27,000 post burn-in trees.** The color scheme corresponds to five discrete states used in reconstruction: (1) 10.01–17.5 mm (white); (2) 17.51–25 mm (blue); (3) 25.01–40 mm (green); (4) 40.01–55 mm (yellow); (5) >55.01 mm (black). Red indicates the fraction of trees for which that node is not present; nodes with equivocal reconstructions are indicated in grey. The three major clades of puddle frogs (A–C) are indicated.
(TIF)

Appendix S1 *BEAST XML file.
(XML)

Table S1 **Specimens examined, including snout-vent length (SVL) and pedal webbing.**
(DOC)

Table S2 **Known reproductive modes of species included in this study.**
(DOC)
