# Supplementary figures and images for: Terrestrialization, Miniaturization and Rates of Diversification in African Puddle Frogs (Anura: Phrynobatrachidae)
Source: PLoS One. 2012 Apr 10;7(4):e35118. doi: 10.1371/journal.pone.0035118 (PMC3325629; doi:10.1371/journal.pone.0035118)

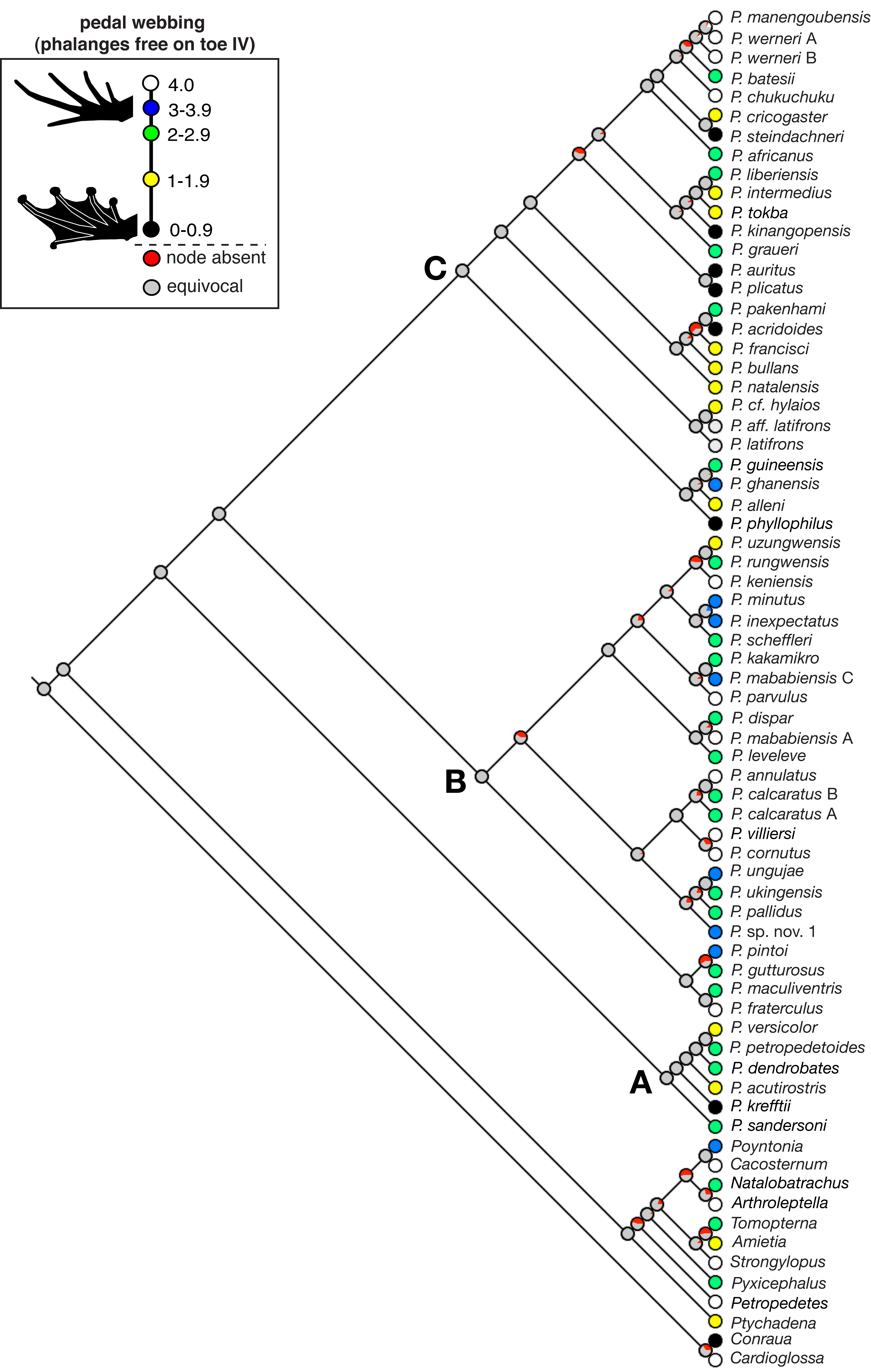

Supplement: Figure S1 — Evolution of pedal webbing. Ancestral state reconstructions of the extent of webbing (minimum number of phalanges free on toe IV) are mapped on each node using using maximum likelihood (ML) in Mesquite on a set of 27,000 post burn-in trees. Number of phalanges free of webbing on toe IV are allocated among five bins: (1) webbing absent, 4 phalanges free (white); (2) webbing rudimentary, 3–3.9 phalanges free (blue); (3) webbing moderate, 2–2.9 phalanges free (green); (4) webbing moderate, 1–1.9 phalanges free (yellow); (5) webbing extensive, 0–0.9 phalanges free (black). Red indicates the fraction of trees for which that node is not present; nodes with equivocal reconstructions are indicated in grey. The three major clades of puddle frogs (A–C) are indicated. (TIF) [file pone.0035118.s001.tif]

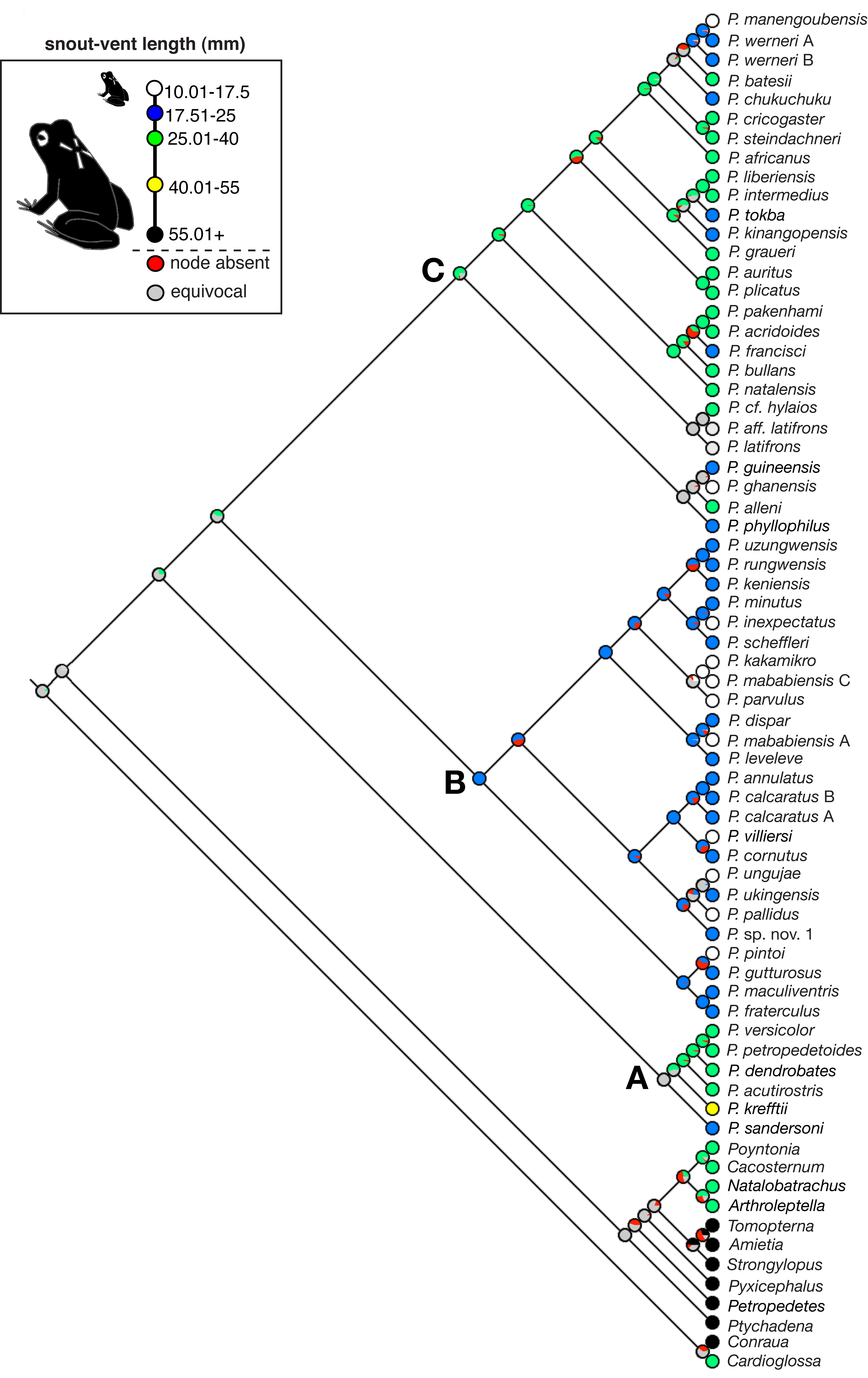

Supplement: Figure S2 — Ancestral state reconstructions for body size (maximum adult snout-vent length) mapped on each node using maximum likelihood (ML) in Mesquite on a set of 27,000 post burn-in trees. The color scheme corresponds to five discrete states used in reconstruction: (1) 10.01–17.5 mm (white); (2) 17.51–25 mm (blue); (3) 25.01–40 mm (green); (4) 40.01–55 mm (yellow); (5) >55.01 mm (black). Red indicates the fraction of trees for which that node is not present; nodes with equivocal reconstructions are indicated in grey. The three major clades of puddle frogs (A–C) are indicated. (TIF) [file pone.0035118.s002.tif]
