# Supplementary material for: Terrestrialization, Miniaturization and Rates of Diversification in African Puddle Frogs (Anura: Phrynobatrachidae)
Source: PLoS One. 2012 Apr 10;7(4):e35118. doi: 10.1371/journal.pone.0035118 (PMC3325629; doi:10.1371/journal.pone.0035118)
Supplement: Table S1 — Specimens examined, including snout-vent length (SVL) and pedal webbing. (DOC) [file pone.0035118.s004.doc]

Table S1. Specimens examined, including snout-vent length (SVL), pedal webbing, carpal state and instances of phalangeal loss.

| **Identification** | **Specimen #** | **Locality** | **SVL** | **Pedal webbing: phalanges free on pedal digit IV** | **Source** |
| --- | --- | --- | --- | --- | --- |
| *Amietia angolensis* | MCZ A-138533 | Tanzania | 90.4 | 2 | This study |
| *Amietia angolensis* | MCZ A-138537 | Tanzania | 60.9 | 2 | This study |
| *Amietia angolensis* | MCZ A-138540 | Tanzania | 60.5 | 2 | This study |
| *Amietia angolensis* | MCZ A-138541 | Tanzania | 77.0 | 2 | This study |
| *Amietia angolensis* | MCZ A-138542 | Tanzania | 65.4 | 2 | This study |
| *Amietia angolensis* | MCZ A-138563 | Tanzania | 66.0 | 2 | This study |
| *Amietia fuscigula* | - | - | 125.0 | - | Channing, 2001 |
| *Amietia vertebralis* | - | - | 145.0 | - | Channing, 2001 |
| *Arthroleptella hewitti* | CAS 156518 | South Africa | 25.2 | - | This study |
| *Arthroleptella hewitti* | MCZ A-26478 | South Africa | 25.4 | 4 | This study |
| *Arthroleptella hewitti* | MCZ A-36727 | South Africa | 23.2 | 4 | This study |
| *Arthroleptella hewitti* | MCZ A-36728 | South Africa | 22.9 | 4 | This study |
| *Arthroleptella hewitti* | MCZ A-36729 | South Africa | 25.8 | 4 | This study |
| *Arthroleptella lightfooti* | CAS 85899 | South Africa | 15.9 | - | This study |
| *Arthroleptella lightfooti* | MCZ A-19931 | South Africa | 20.0 | 4 | This study |
| *Arthroleptella lightfooti* | MCZ A-19932 | South Africa | 18.9 | 4 | This study |
| *Arthroleptella lightfooti* | MCZ A-28312 | South Africa | 13.5 | 4 | This study |
| *Arthroleptella lightfooti* | MCZ A-28313 | South Africa | 14.0 | 4 | This study |
| *Arthroleptella lightfooti* | MCZ A-28314 | South Africa | 12.9 | 4 | This study |
| *Cacosternum boettgeri* | MCZ A-20822 | South Africa | 15.0 | 4 | This study |
| *Cacosternum boettgeri* | MCZ A-20823 | South Africa | 15.9 | 4 | This study |
| *Cacosternum boettgeri* | MCZ A-20824 | South Africa | 16.9 | 4 | This study |
| *Cacosternum boettgeri* | MCZ A-20825 | South Africa | 15.3 | 4 | This study |
| *Cacosternum boettgeri* | MCZ A-24495 | South Africa | 22.2 | 4 | This study |
| *Cacosternum capense* | - | South Africa | 39.0 | - | Channing, 2001 |
| *Cardioglossa gracilis* | MCZ A-136796 | Cameroon | 32.5 | 4 | This study |
| *Cardioglossa gracilis* | MCZ A-137931 | Cameroon | 29.8 | 4 | This study |
| *Cardioglossa gracilis* | MCZ A-137932 | Cameroon | 31.2 | 4 | This study |
| *Cardioglossa gracilis* | MCZ A-137933 | Cameroon | 28.9 | 4 | This study |
| *Cardioglossa gracilis* | MCZ A-137934 | Cameroon | 29.8 | 4 | This study |
| *Cardioglossa gracilis* | MCZ A-137936 | Cameroon | 28.8 | - | This study |
| *Cardioglossa leucomystax* | - | Cameroon | 39.7 | - | Blackburn, 2009 |
| *Conraua crassipes* | MCZ A-5581 | Cameroon | 53.9 | 0 | This study |
| *Conraua crassipes* | MCZ A-23247 | Cameroon | 55.0 | 0 | This study |
| *Conraua crassipes* | MCZ A-23248 | Cameroon | 52.7 | 0 | This study |
| *Conraua crassipes* | MCZ A-136807 | Cameroon | 34.6 | 0 | This study |
| *Conraua goliath* | - | - | 320.0 | - | Sabater-Pi, 1985 |
| *Natalobatrachus bonebergi* | ES 546 | South Africa | 26.1 | - | This study |
| *Natalobatrachus bonebergi* | ES 547 | South Africa | 27.1 | - | This study |
| *Natalobatrachus bonebergi* | MCZ A-100013 | South Africa | 26.3 | 3 | This study |
| *Natalobatrachus bonebergi* | MCZ A-100014 | South Africa | 26.7 | 3 | This study |
| *Natalobatrachus bonebergi* | MCZ A-100015 | South Africa | 24.1 | 3 | This study |
| *Natalobatrachus bonebergi* | MCZ A-36738 | South Africa | 27.7 | 3 | This study |
| *Natalobatrachus bonebergi* | MCZ A-36739 | South Africa | 24.0 | 3 | This study |
| *Natalobatrachus bonebergi* | - | South Africa | 37.0 | - | Channing, 2001 |
| *Petropedetes martiensseni* | CAS 168628 | Tanzania | 50.9 | - | This study |
| *Petropedetes martiensseni* | MCZ A-12822 | Tanzania | 30.7 | 4 | This study |
| *Petropedetes martiensseni* | MCZ A-12824 | Tanzania | 40.8 | 4 | This study |
| *Petropedetes martiensseni* | MCZ A-138520 | Tanzania | 56.0 | 4 | This study |
| *Petropedetes martiensseni* | MCZ A-138521 | Tanzania | 55.6 | 4 | This study |
| *Petropedetes martiensseni* | MCZ A-138522 | Tanzania | 35.9 | 4 | This study |
| *Petropedetes martiensseni* | MCZ A-25381 | Tanzania | 62.7 | 4 | This study |
| *Petropedetes yakusini* | - | Tanzania | 73.0 | - | Channing, 2001 |
| *Phrynobatrachus accraensis* | MVZ 245114 | Ghana | 23.2 | 2 | This study |
| *Phrynobatrachus accraensis* | MVZ 245120 | Ghana | 22.0 | 2 | This study |
| *Phrynobatrachus accraensis* | MVZ 245150 | Ghana | 20.3 | 2 | This study |
| *Phrynobatrachus accraensis* | MVZ 245154 | Ghana | 18.8 | 2 | This study |
| *Phrynobatrachus accraensis* | ZMB 28344 | Ghana | 20.5 | 2.5 | This study |
| *Phrynobatrachus acridoides* | CAS 148377 | Somalia | 24.4 | - | This study |
| *Phrynobatrachus acridoides* | MCZ A-138213 | Tanzania | 25.5 | 2 | This study |
| *Phrynobatrachus acridoides* | MCZ A-138214 | Tanzania | 23.6 | 2 | This study |
| *Phrynobatrachus acridoides* | MCZ A-138215 | Tanzania | 22.4 | 2 | This study |
| *Phrynobatrachus acridoides* | MCZ A-138216 | Tanzania | 23.0 | 1 | This study |
| *Phrynobatrachus acridoides* | MCZ A-138216 | Tanzania | 22.2 | 1 | This study |
| *Phrynobatrachus acridoides* | MCZ A-138287 | Tanzania | 23.5 | 2 | This study |
| *Phrynobatrachus acutirostris* | CAS-SUA 13011 | Dem. Rep. Congo | 40.0 | - | This study |
| *Phrynobatrachus acutirostris* | EGB 1314 | Dem. Rep. Congo | 30.3 | 3.5 | This study |
| *Phrynobatrachus acutirostris* | EGB 1317 | Dem. Rep. Congo | 26.4 | 3.5 | This study |
| *Phrynobatrachus acutirostris* | MCZ A-26502 | Dem. Rep. Congo | 39.8 | 2 | This study |
| *Phrynobatrachus acutirostris* | MCZ A-26503 | Dem. Rep. Congo | 37.0 | 2 | This study |
| *Phrynobatrachus aff. accraensis* | BM 2005.1185 | Tanzania | 24.3 | - | This study |
| *Phrynobatrachus aff. accraensis* | BM 2005.1203 | Tanzania | 12.2 | - | This study |
| *Phrynobatrachus aff. accraensis* | BM 2005.1206 | Tanzania | 18.7 | - | This study |
| *Phrynobatrachus aff. accraensis* | BM 2005.1217 | Tanzania | 19.8 | - | This study |
| *Phrynobatrachus africanus* | CAS 153803 | Cameroon | 25.4 | - | This study |
| *Phrynobatrachus africanus* | MCZ A-136800 | Cameroon | 30.3 | 3 | This study |
| *Phrynobatrachus africanus* | MCZ A-136945 | Cameroon | 30.4 | 3.5 | This study |
| *Phrynobatrachus africanus* | MCZ A-138103 | Cameroon | 26.4 | 3.5 | This study |
| *Phrynobatrachus africanus* | MCZ A-138142 | Cameroon | 17.8 | 3.5 | This study |
| *Phrynobatrachus africanus* | MCZ A-138143 | Cameroon | 21.5 | 3.5 | This study |
| *Phrynobatrachus africanus* | MCZ A-20976 | Cameroon | 20.1 | 3 | This study |
| *Phrynobatrachus alleni* | MCZ A-138849 | Liberia | 26.5 | - | This study |
| *Phrynobatrachus alleni* | MCZ A-138850 | Liberia | 21.9 | 3 | This study |
| *Phrynobatrachus alleni* | MCZ A-138855 | Liberia | 25.5 | 2 | This study |
| *Phrynobatrachus alleni* | MCZ A-138857 | Liberia | 25.0 | 2 | This study |
| *Phrynobatrachus alleni* | MCZ A-138862 | Liberia | 29.7 | 2 | This study |
| *Phrynobatrachus annulatus* | - | - | 25.0 | 4 | Rödel and Ernst, 2002 (N=3); Perret, 1988 |
| *Phrynobatrachus auritus* | MCZ A-138094 | Cameroon | 33.7 | 1 | This study |
| *Phrynobatrachus auritus* | MCZ A-138095 | Cameroon | 30.9 | 1 | This study |
| *Phrynobatrachus auritus* | MCZ A-138096 | Cameroon | 34.7 | 1 | This study |
| *Phrynobatrachus auritus* | MCZ A-138097 | Cameroon | 35.6 | 1 | This study |
| *Phrynobatrachus auritus* | MCZ A-138098 | Cameroon | 33.0 | 1 | This study |
| *Phrynobatrachus batesii* | MCZ A-136783 | Cameroon | 31.0 | 3.5 | This study |
| *Phrynobatrachus batesii* | MCZ A-136784 | Cameroon | 26.5 | 3 | This study |
| *Phrynobatrachus batesii* | MCZ A-136793 | Cameroon | 30.0 | 3 | This study |
| *Phrynobatrachus batesii* | MCZ A-5601 | Cameroon | 25.1 | 4 | This study |
| *Phrynobatrachus batesii* | MCZ A-5602 | Cameroon | 22.6 | 4 | This study |
| *Phrynobatrachus batesii* | MCZ A-5603 | Cameroon | 25.4 | 4 | This study |
| *Phrynobatrachus bequaerti* | CAS 98154 | Dem. Rep. Congo | 22.1 | - | This study |
| *Phrynobatrachus bequaerti* | MCZ A-14752 | Dem. Rep. Congo | 22.2 | 3 | This study |
| *Phrynobatrachus bequaerti* | MCZ A-14753 | Dem. Rep. Congo | 16.9 | 3 | This study |
| *Phrynobatrachus bequaerti* | MCZ A-14755 | Dem. Rep. Congo | 20.8 | 3 | This study |
| *Phrynobatrachus bequaerti* | MCZ A-14756 | Dem. Rep. Congo | 19.1 | 3 | This study |
| *Phrynobatrachus bequaerti* | MCZ A-49805 | Dem. Rep. Congo | 17.1 | 3.5 | This study |
| *Phrynobatrachus bequaerti* | MCZ A-49816 | Dem. Rep. Congo | 22.1 | 3.5 | This study |
| *Phrynobatrachus bullans* | AAU A2008-032 | Ethiopia | 25.1 | 2 | This study |
| *Phrynobatrachus bullans* | MTSN 5864 | Tanzania | 25.7 | 2.5 | This study |
| *Phrynobatrachus bullans* | MVZ 234151 | Tanzania | 26.0 | 3.5 | This study |
| *Phrynobatrachus bullans* | MVZ 238716 | Kenya | 23.0 | 3 | This study |
| *Phrynobatrachus bullans* | - | Tanzania | 27.2 | 1.75 | Crutsinger *et al.,* 2007 |
| *Phrynobatrachus calcaratus* | CAS 146090 | Ghana | 17.7 | - | This study |
| *Phrynobatrachus calcaratus* | MCZ A-20959 | Cameroon | 15.5 | 4 | This study |
| *Phrynobatrachus calcaratus* | MCZ A-20960 | Cameroon | 16.9 | 4 | This study |
| *Phrynobatrachus calcaratus* | MCZ A-46775 | Dem. Rep. Congo | 17.6 | 3 | This study |
| *Phrynobatrachus calcaratus* | MCZ A-46776 | Dem. Rep. Congo | 15.3 | 3 | This study |
| *Phrynobatrachus calcaratus* | MVZ 245139 | Ghana | 19.7 | 4 | This study |
| *Phrynobatrachus calcaratus* | MVZ 245140 | Ghana | 25.0 | 4 | This study |
| *Phrynobatrachus chukuchuku* | MCZ A-138124 | Cameroon | 16.1 | 4 | This study |
| *Phrynobatrachus chukuchuku* | MCZ A-138126 | Cameroon | 18.1 | 4 | This study |
| *Phrynobatrachus chukuchuku* | MCZ A-138127 | Cameroon | 15.7 | 4 | This study |
| *Phrynobatrachus chukuchuku* | MCZ A-138128 | Cameroon | 18.9 | 4 | This study |
| *Phrynobatrachus chukuchuku* | MCZ A-138130 | Cameroon | 18.1 | 4 | This study |
| *Phrynobatrachus cornutus* | CAS 199268 | Cameroon | 18.9 | 4 | This study |
| *Phrynobatrachus cornutus* | CAS 207799 | Equatorial Guinea: Bioko | 19.1 | 4 | This study |
| *Phrynobatrachus cornutus* | CAS 207877 | Equatorial Guinea: Bioko | 14.0 | 4 | This study |
| *Phrynobatrachus cornutus* | MCZ A-136837 | Cameroon | 18.7 | 4 | This study |
| *Phrynobatrachus cricogaster* | CAS 158975 | Cameroon | 23.7 | - | This study |
| *Phrynobatrachus cricogaster* | MCZ A-138030 | Cameroon | 19.8 | 3 | This study |
| *Phrynobatrachus cricogaster* | MCZ A-31572 | Cameroon | 22.0 | 1 | This study |
| *Phrynobatrachus cricogaster* | MCZ A-31573 | Cameroon | 23.8 | 1 | This study |
| *Phrynobatrachus cricogaster* | - | Cameroon | 32.0 | 2.5-3 | Perret, 1966 (N=54) |
| *Phrynobatrachus dendrobates* | CAS 145294 | Zaire | 27.0 | - | This study |
| *Phrynobatrachus dendrobates* | MCZ A-14653 | Zaire | 33.2 | 3 | This study |
| *Phrynobatrachus dendrobates* | MCZ A-14655 | Zaire | 34.0 | 3 | This study |
| *Phrynobatrachus dendrobates* | MCZ A-14660 | Zaire | 33.4 | 3 | This study |
| *Phrynobatrachus dendrobates* | MCZ A-14666 | Zaire | 33.2 | 3 | This study |
| *Phrynobatrachus dendrobates* | MCZ A-14667 | Zaire | 37.0 | 3 | This study |
| *Phrynobatrachus dendrobates* | MCZ A-14673 | Zaire | 34.2 | 3 | This study |
| *Phrynobatrachus dispar* | CAS 219080 | Sao Tome and Principe: Principe | 16.9 | 3 | This study |
| *Phrynobatrachus dispar* | CAS 219081 | Sao Tome and Principe: Principe | 17.1 | 3 | This study |
| *Phrynobatrachus dispar* | CAS 219082 | Sao Tome and Principe: Principe | 16.8 | 3 | This study |
| *Phrynobatrachus dispar* | CAS 219083 | Sao Tome and Principe: Principe | 15.6 | 3 | This study |
| *Phrynobatrachus dispar* | CAS 219084 | Sao Tome and Principe: Principe | 16.8 | 3 | This study |
| *Phrynobatrachus dispar* | CAS 219103 | Sao Tome and Principe: Principe | 15.6 | - | This study |
| *Phrynobatrachus dispar* | CAS 219389 | Sao Tome and Principe: Principe | 23.0 | - | This study |
| *Phrynobatrachus francisci* | MCZ A-138847 | Nigeria | 16.8 | - | This study |
| *Phrynobatrachus francisci* | MVZ 234836 | Cameroon | 19.2 | 2 | This study |
| *Phrynobatrachus francisci* | BM 1947.2.30.29 | Nigeria | 15.0 | - | Boulenger, 1912 |
| *Phrynobatrachus francisci* | - | - | 21.5 | 3 | Lamotte and Xavier, 1966 (N=47) |
| *Phrynobatrachus fraterculus* | MCZ A-12032 | Liberia | 18.1 | 4 | This study |
| *Phrynobatrachus fraterculus* | - | - | 25.0 | 4 | Rödel and Ernst, 2002 (N=4); Guibé and Lamotte, 1963 (N=153) |
| *Phrynobatrachus ghanensis* | ZMUC R074712 | Ghana | 13.8 | 3.75 | Schiøtz, 1964 |
| *Phrynobatrachus ghanensis* | ZMUC R074707 | Ghana | 13.2 | 3.75 | Schiøtz, 1964 |
| *Phrynobatrachus graueri* | CAS 141589 | Kenya | 19.1 | - | This study |
| *Phrynobatrachus graueri* | UTACV A-58276 | Rwanda | 18.7 | 3.5 | This study |
| *Phrynobatrachus graueri* | UTACV A-58277 | Rwanda | 19.0 | 3.5 | This study |
| *Phrynobatrachus graueri* | UTACV A-58278 | Rwanda | 19.3 | 3 | This study |
| *Phrynobatrachus graueri* | UTACV A-58279 | Rwanda | 18.6 | 3 | This study |
| *Phrynobatrachus graueri* | UTACV A-58280 | Rwanda | 25.8 | 3.5 | This study |
| *Phrynobatrachus graueri* | MCZ A-12839 | Kenya | 20.2 | 4 | This study |
| *Phrynobatrachus graueri* | MCZ A-25460 | Uganda | 20.0 | 3 | This study |
| *Phrynobatrachus graueri* | MCZ A-25461 | Uganda | 26.3 | 3 | This study |
| *Phrynobatrachus guineensis* | - | - | 22.0 | 3 | Guibé and Lamotte, 1961 (N=24); Rödel and Ernst, 2002 (N=16) |
| *Phrynobatrachus gutturosus* | - | - | 23.0 | 3-4 | Chabanaud, 1921; Guibé and Lamotte, 1963 (N=33); Rödel and Ernst, 2002 (N=13) |
| *Phrynobatrachus gutturosus* | MCZ A-12836 | Liberia | 18.1 | 4 | This study |
| *Phrynobatrachus hylaios* | AMNH 8976 | Dem. Rep. Congo | 16.0 | 3 | This study |
| *Phrynobatrachus hylaios* | AMNH 8978 | Dem. Rep. Congo | 14.8 | 4 | This study |
| *Phrynobatrachus hylaios* | - | Cameroon | 22.0 | 4 | Perret, 1966 (N=123) |
| *Phrynobatrachus hylaios* (cf.) | MCZ A-136011 | Rep. of Congo | 25.7 | 2 | This study |
| *Phrynobatrachus hylaios* (cf.) | MCZ A-136014 | Rep. of Congo | 24.2 | 2 | This study |
| *Phrynobatrachus hylaios* (cf.) | MCZ A-136015 | Rep. of Congo | 24.1 | 2 | This study |
| *Phrynobatrachus hylaios* (cf.) | MCZ A-136016 | Rep. of Congo | 26.1 | 2 | This study |
| *Phrynobatrachus hylaios* (cf.) | MCZ A-136017 | Rep. of Congo | 24.6 | 2 | This study |
| *Phrynobatrachus inexpectatus* | AAU A2008-043 | Ethiopia | 15.7 | 3.75 | This study |
| *Phrynobatrachus inexpectatus* | AAU A2008-044 | Ethiopia | 16.4 | 3.75 | This study |
| *Phrynobatrachus inexpectatus* | AAU A2008-045 | Ethiopia | 16.7 | 3.75 | This study |
| *Phrynobatrachus intermedius* | ZMB 71538 | Côte d’Ivoire | 27.8 | 1.5 | Rödel *et al.*, 2009a |
| *Phrynobatrachus intermedius* | ZMB 71539 | Côte d’Ivoire | 22.8 | - | Rödel *et al.*, 2009a |
| *Phrynobatrachus irangi* | CAS 158966 | Kenya | 46.5 | - | This study |
| *Phrynobatrachus kakamikro* | MCZ A-20439 | Tanzania | 12.0 | 3.75 | This study |
| *Phrynobatrachus kakamikro* | - | - | 19.0 | 3 | Schick *et al.*, 2009 (N=4) |
| *Phrynobatrachus keniensis* | MCZ A-138868 | Kenya | 19.7 | 4 | This study |
| *Phrynobatrachus keniensis* | MCZ A-16114 | Kenya | 22.0 | 4 | This study |
| *Phrynobatrachus keniensis* | MCZ A-19870 | Kenya | 22.4 | 4 | This study |
| *Phrynobatrachus keniensis* | MCZ A-19871 | Kenya | 18.5 | 4 | This study |
| *Phrynobatrachus keniensis* | MCZ A-19872 | Kenya | 19.5 | 4 | This study |
| *Phrynobatrachus keniensis* | MCZ A-19873 | Kenya | 17.6 | 4 | This study |
| *Phrynobatrachus keniensis* | MVZ 226260 | Kenya | 19.5 | 4 | This study |
| *Phrynobatrachus kinangopensis* | CAS 152381 | Kenya | 21.1 | - | This study |
| *Phrynobatrachus kinangopensis* | MCZ A-20457 | Kenya | 19.9 | 1 | This study |
| *Phrynobatrachus kinangopensis* | MCZ A-20461 | Kenya | 22.8 | 3 | This study |
| *Phrynobatrachus kinangopensis* | MCZ A-20462 | Kenya | 18.8 | 2 | This study |
| *Phrynobatrachus kinangopensis* | MCZ A-25424 | Kenya | 13.9 | 2 | This study |
| *Phrynobatrachus kinangopensis* | MCZ A-25425 | Kenya | 17.7 | 2 | This study |
| *Phrynobatrachus krefftii* | CAS 168514 | Tanzania | 38.1 | - | This study |
| *Phrynobatrachus krefftii* | MCZ A-138299 | Tanzania | 39.2 | 2 | This study |
| *Phrynobatrachus krefftii* | MCZ A-138302 | Tanzania | 42.9 | 1 | This study |
| *Phrynobatrachus krefftii* | MCZ A-138304 | Tanzania | 40.4 | 3 | This study |
| *Phrynobatrachus krefftii* | MCZ A-138306 | Tanzania | 43.8 | 2 | This study |
| *Phrynobatrachus krefftii* | MCZ A-138307 | Tanzania | 24.9 | 2 | This study |
| *Phrynobatrachus krefftii* | MCZ A-138310 | Tanzania | 33.2 | 1 | This study |
| *Phrynobatrachus krefftii* | MCZ A-138311 | Tanzania | 35.6 | 2 | This study |
| *Phrynobatrachus krefftii* | MCZ A-138313 | Tanzania | 40.3 | 2 | This study |
| *Phrynobatrachus krefftii* | MCZ A-138316 | Tanzania | 38.6 | 1 | This study |
| *Phrynobatrachus krefftii* | MCZ A-138364 | Tanzania | 29.6 | 2 | This study |
| *Phrynobatrachus leveleve* | CAS 218893 | São Tomé and Príncipe: São Tomé | 18.3 | 4 | This study |
| *Phrynobatrachus leveleve* | CAS 218894 | São Tomé and Príncipe: São Tomé | 13.7 | 4 | This study |
| *Phrynobatrachus leveleve* | CAS 218895 | São Tomé and Príncipe: São Tomé | 15.2 | - | This study |
| *Phrynobatrachus leveleve* | CAS 218897 | São Tomé and Príncipe: São Tomé | 14.4 | 3 | This study |
| *Phrynobatrachus leveleve* | CAS 218898 | São Tomé and Príncipe: São Tomé | 13.4 | 3.5 | This study |
| *Phrynobatrachus leveleve* | CAS 218899 | São Tomé and Príncipe: São Tomé | 13.1 | 3.5 | This study |
| *Phrynobatrachus leveleve* | CAS 218998 | São Tomé and Príncipe: São Tomé | 22.3 | - | This study |
| *Phrynobatrachus liberiensis* | - | - | 35.5 | 3 | Rödel and Ernst, 2002 (N=134); Guibé and Lamotte, 1963 (N=31) |
| *Phrynobatrachus liberiensis* | MCZ A-11993 | Liberia | 31.8 | 3 | This study |
| *Phrynobatrachus liberiensis* | MCZ A-21409 | Liberia | 31.8 | 3 | This study |
| *Phrynobatrachus mababiensis* A | CAS 196596 | Zambia | 16.1 | - | This study |
| *Phrynobatrachus mababiensis* A | MCZ A-17717 | Bostwana | 11.7 | 4 | This study |
| *Phrynobatrachus mababiensis* A | MCZ A-17718 | Bostwana | 11.7 | 4 | This study |
| *Phrynobatrachus mababiensis* C | MCZ A-138319 | Tanzania | 15.9 | 4 | This study |
| *Phrynobatrachus mababiensis* C | MCZ A-138321 | Tanzania | 16.2 | 3.5 | This study |
| *Phrynobatrachus maculiventris* | MCZ A-12034 | Liberia | 20.6 | 4 | This study |
| *Phrynobatrachus maculiventris* | ZMB 71592 | Guinea | 17.7 | 3 | Rödel *et. al.,*2009b |
| *Phrynobatrachus maculiventris* | ZMB 71593 | Guinea | 18.2 | 3 | Rödel *et. al.,*2009b |
| *Phrynobatrachus maculiventris* | - |  | 25.0 | 4 | Guibé and Lamotte, 1963 (N=25) |
| *Phrynobatrachus manengoubensis* | MCZ A-136923 | Cameroon | 16.8 | 4 | This study |
| *Phrynobatrachus manengoubensis* | MCZ A-136924 | Cameroon | 16.2 | 4 | This study |
| *Phrynobatrachus manengoubensis* | MCZ A-136925 | Cameroon | 15.3 | 4 | This study |
| *Phrynobatrachus manengoubensis* | MCZ A-136938 | Cameroon | 17.3 | - | This study |
| *Phrynobatrachus manengoubensis* | MCZ A-138036 | Cameroon | 15.7 | 4 | This study |
| *Phrynobatrachus manengoubensis* | MNHN 1939-113 | Cameroon | 14.2 | 4 | This study |
| *Phrynobatrachus minutus* | AAU A2008-062 | Ethiopia | 19.0 | 3.5 | This study |
| *Phrynobatrachus minutus* | AAU A2008-063 | Ethiopia | 17.9 | 3.5 | This study |
| *Phrynobatrachus minutus* | AAU A2008-064 | Ethiopia | 19.1 | 3.5 | This study |
| *Phrynobatrachus minutus* | AAU A2008-065 | Ethiopia | 18.2 | 3.5 | This study |
| *Phrynobatrachus minutus* | AAU A2008-102 | Ethiopia | 19.0 | 3.5 | This study |
| *Phrynobatrachus minutus* | AAU A2008-108 | Ethiopia | 18.0 | 3.5 | This study |
| *Phrynobatrachus minutus* | AAU A2008-112 | Ethiopia | 15.5 | 3.5 | This study |
| *Phrynobatrachus natalensis* | AAU A2008-117 | Ethiopia | 29.3 | 2.5 | This study |
| *Phrynobatrachus natalensis* | AAU A2008-223 | Ethiopia | 26.3 | 2.5 | This study |
| *Phrynobatrachus natalensis* | AAU A2008-229 | Ethiopia | 30.8 | 2.5 | This study |
| *Phrynobatrachus natalensis* | AAU A2008-231 | Ethiopia | 30.4 | 2.75 | This study |
| *Phrynobatrachus natalensis* | CAS 141564 | Kenya | 30.3 | - | This study |
| *Phrynobatrachus natalensis* | ES 283 | South Africa | 29.8 | - | This study |
| *Phrynobatrachus natalensis* | ES 287 | South Africa | 31.6 | - | This study |
| *Phrynobatrachus natalensis* | MCZ A-138082 | Cameroon | 25.7 | 3 | This study |
| *Phrynobatrachus natalensis* | MCZ A-138083 | Cameroon | 26.7 | 3 | This study |
| *Phrynobatrachus natalensis* | MCZ A-138340 | Tanzania | 25.4 | 3 | This study |
| *Phrynobatrachus natalensis* | MCZ A-138347 | Tanzania | 20.9 | 2 | This study |
| *Phrynobatrachus natalensis* | MVZ 234059 | Uganda | 24.9 | 3.5 | This study |
| *Phrynobatrachus natalensis* | MVZ 245160 | Ghana | 30.2 | 2 | This study |
| *Phrynobatrachus natalensis* | MVZ 245161 | Ghana | 33.4 | 2 | This study |
| *Phrynobatrachus pakenhami* | MCZ 138269 | Tanzania | 24.0 | 3 | This study |
| *Phrynobatrachus pakenhami* | MCZ A- 138262 | Tanzania | 19.0 | - | This study |
| *Phrynobatrachus pakenhami* | MCZ A-138238 | Tanzania | 27.5 | 3 | This study |
| *Phrynobatrachus pakenhami* | MCZ A-138258 | Tanzania | 28.8 | 3 | This study |
| *Phrynobatrachus pakenhami* | MCZ A-138265 | Tanzania | 28.4 | 3 | This study |
| *Phrynobatrachus pakenhami* | MCZ A-138267 | Tanzania | 26.9 | 3 | This study |
| *Phrynobatrachus pakenhami* | MCZ A-138277 | Tanzania | 25.2 | - | This study |
| *Phrynobatrachus pallidus* | MVZ 234153 | Kenya | 17.0 | 4 | This study |
| *Phrynobatrachus pallidus* | LIVM 1998.46.36 | Tanzania | 17.5 | 3 | Pickersgill, 2007 |
| *Phrynobatrachus parkeri* | CAS 98168 | Zaire | 24.4 | - | This study |
| *Phrynobatrachus parkeri* | CAS 98168 | Zaire | 24.1 | - | This study |
| *Phrynobatrachus parkeri* | MCZ 217151 | Zaire | 20.1 | 3 | This study |
| *Phrynobatrachus parkeri* | MCZ 217152 | Zaire | 19.3 | 3 | This study |
| *Phrynobatrachus parkeri* | MCZ 26515 | Zaire | 20.9 | 3 | This study |
| *Phrynobatrachus parvulus* | CAS 145258 | Zaire | 16.5 | - | This study |
| *Phrynobatrachus parvulus* | MCZ A-137077 | Malawi | 16.1 | 4 | This study |
| *Phrynobatrachus parvulus* | MCZ A-137103 | Malawi | 13.5 | 4 | This study |
| *Phrynobatrachus parvulus* | MCZ A-137112 | Malawi | 13.1 | 4 | This study |
| *Phrynobatrachus parvulus* | MCZ A-137120 | Malawi | 16.6 | 4 | This study |
| *Phrynobatrachus perpalmatus* | CAS 98156 | Dem. Rep. Congo | 23.6 | - | This study |
| *Phrynobatrachus perpalmatus* | MCZ A-107519 | Mozambique | 25.0 | 2 | This study |
| *Phrynobatrachus perpalmatus* | MCZ A-107523 | Mozambique | 20.1 | 2 | This study |
| *Phrynobatrachus perpalmatus* | MCZ A-107524 | Mozambique | 19.1 | 2 | This study |
| *Phrynobatrachus perpalmatus* | MCZ A-107525 | Mozambique | 23.6 | 2 | This study |
| *Phrynobatrachus perpalmatus* | MCZ A-107526 | Mozambique | 24.7 | 2 | This study |
| *Phrynobatrachus perpalmatus* | MCZ A-21750 | Dem. Rep. Congo | 28.3 | 1 | This study |
| *Phrynobatrachus petropedetoides* | MCZ A-138201 | Dem. Rep. Congo | 27.3 | 3 | This study |
| *Phrynobatrachus petropedetoides* | MCZ A-17534 | Dem. Rep. Congo | 35.6 | 3 | This study |
| *Phrynobatrachus petropedetoides* | MCZ A-17535 | Dem. Rep. Congo | 25.2 | 3.5 | This study |
| *Phrynobatrachus petropedetoides* | MCZ A-49635 | Dem. Rep. Congo | 33.9 | 3 | This study |
| *Phrynobatrachus petropedetoides* | MCZ A-49639 | Dem. Rep. Congo | 32.4 | 3 | This study |
| *Phrynobatrachus petropedetoides* | MCZ A-49643 | Dem. Rep. Congo | 32.0 | 3 | This study |
| *Phrynobatrachus petropedetoides* | MCZ A-49654 | Dem. Rep. Congo | 35.3 | 3 | This study |
| *Phrynobatrachus petropedetoides* | MCZ A-49668 | Dem. Rep. Congo | 29.2 | 3 | This study |
| *Phrynobatrachus phyllophilus* | - | Guinea | 23.0 | 3 | Rödel and Ernst, 2002 (N=131) |
| *Phrynobatrachus pintoi* | ZMB 70689 | Guinea | 10.2 | 3.75 | Hillers et. al., 2008 |
| *Phrynobatrachus plicatus* | CAS 136298 | Ghana | 33.5 | - | This study |
| *Phrynobatrachus plicatus* | MCZ A-21753 | Dem. Rep. Congo | 28.6 | 2 | This study |
| *Phrynobatrachus plicatus* | MCZ A-21754 | Dem. Rep. Congo | 30.5 | 1 | This study |
| *Phrynobatrachus plicatus* | MCZ A-2609 | Cameroon | 22.6 | 2 | This study |
| *Phrynobatrachus plicatus* | MVZ 245164 | Ghana | 35.3 | 3 | This study |
| *Phrynobatrachus plicatus* | MVZ 245165 | Ghana | 38.9 | 3 | This study |
| *Phrynobatrachus rungwensis* | BM 2005.858 | Tanzania | 16.6 | 2.5 | Specimen |
| *Phrynobatrachus rungwensis* | BM 2005.859 | Tanzania | 21.6 | 2.5 | Specimen |
| *Phrynobatrachus rungwensis* | MCZ A-21698 | Dem. Rep. Congo | 20.3 | 3 | This study |
| *Phrynobatrachus rungwensis* | MCZ A-100737 | Dem. Rep. Congo | 21.3 | 3 | This study |
| *Phrynobatrachus rungwensis* | MCZ A-100738 | Dem. Rep. Congo | 18.4 | 3 | This study |
| *Phrynobatrachus rungwensis* | MCZ A-100739 | Dem. Rep. Congo | 17.6 | 3 | This study |
| *Phrynobatrachus rungwensis* | MCZ A-21694 | Dem. Rep. Congo | 21.3 | 3 | This study |
| *Phrynobatrachus rungwensis* | MCZ A-21695 | Dem. Rep. Congo | 21.9 | 3 | This study |
| *Phrynobatrachus rungwensis* | MCZ A-21697 | Dem. Rep. Congo | 20.6 | 3 | This study |
| *Phrynobatrachus rungwensis* | MCZ A-21700 | Dem. Rep. Congo | 17.0 | 3 | This study |
| *Phrynobatrachus sandersoni* | MCZ A- 136790 | Cameroon | 21.2 | 3 | This study |
| *Phrynobatrachus sandersoni* | MCZ A- 136791 | Cameroon | 23.0 | 3 | This study |
| *Phrynobatrachus sandersoni* | MCZ A- 136794 | Cameroon | 23.0 | 3 | This study |
| *Phrynobatrachus scheffleri* | MVZ 234062 | Kenya | 18.0 | 3.5 | This study |
| *Phrynobatrachus scheffleri* | MVZ 234149 | Kenya | 12.4 | 3.5 | This study |
| *Phrynobatrachus scheffleri* | - | - | 19.1 | 3 | Schick *et al*., 2009 (N=67) |
| *Phrynobatrachus sp. nov.* | AAU A2008-348 | Ethiopia | 17.8 | 3.75 | This study |
| *Phrynobatrachus sp. nov.* | AAU A2008-349 | Ethiopia | 17.8 | 3.75 | This study |
| *Phrynobatrachus sp. nov.* | AAU A2008-350 | Ethiopia | 18.3 | 3.5 | This study |
| *Phrynobatrachus sp. nov.* | AAU A2008-351 | Ethiopia | 16.3 | 4 | This study |
| *Phrynobatrachus sp. nov.* | AAU A2008-352 | Ethiopia | 17.0 | 4 | This study |
| *Phrynobatrachus sp. nov.* | AAU A2008-353 | Ethiopia | 15.1 | 3.75 | This study |
| *Phrynobatrachus sp. nov.* | AAU A2008-354 | Ethiopia | 17.0 | 3.5 | This study |
| *Phrynobatrachus steindachneri* | MCZ A-136906 | Cameroon | 36.6 | - | This study |
| *Phrynobatrachus steindachneri* | MCZ A-138064 | Cameroon | 15.1 | 2 | This study |
| *Phrynobatrachus steindachneri* | MCZ A-138115 | Cameroon | 32.1 | - | This study |
| *Phrynobatrachus steindachneri* | MCZ A-138135 | Cameroon | 30.6 | 2 | This study |
| *Phrynobatrachus steindachneri* | MCZ A-138136 | Cameroon | 29.0 | 2 | This study |
| *Phrynobatrachus steindachneri* | MCZ A-138137 | Cameroon | 26.5 | 2 | This study |
| *Phrynobatrachus steindachneri* | MCZ A-138139 | Cameroon | 28.4 | 2 | This study |
| *Phrynobatrachus steindachneri* | MCZ A-138140 | Cameroon | 30.7 | 2 | This study |
| *Phrynobatrachus tokba* | MCZ A- 26901 | Sierra Leone | 12.1 | 4 | This study |
| *Phrynobatrachus tokba* | MCZ A- 26902 | Sierra Leone | 12.7 | 4 | This study |
| *Phrynobatrachus tokba* | MCZ A- 26904 | Sierra Leone | 11.3 | 4 | This study |
| *Phrynobatrachus tokba* | - | - | 20.0 | - | Guibé and Lamotte, 1961 (N=84) |
| *Phrynobatrachus ukingensis* | BM 2005.144 | Tanzania | 17.1 | 3.75 | This study |
| *Phrynobatrachus ukingensis* | CAS 184136 | Kenya | 16.1 | - | This study |
| *Phrynobatrachus ukingensis* | MCZ A-100762 | Malawi | 14.0 | 3 | This study |
| *Phrynobatrachus ukingensis* | MCZ A-105993 | Kenya | 16.3 | 3.5 | This study |
| *Phrynobatrachus ukingensis* | MCZ A-17137 | Tanzania | 18.7 | 4 | This study |
| *Phrynobatrachus ukingensis* | MCZ A-17138 | Tanzania | 14.2 | 4 | This study |
| *Phrynobatrachus ukingensis* | MCZ A-27595 | Malawi | 20.3 | 3 | This study |
| *Phrynobatrachus ukingensis* | MCZ A-27609 | Malawi | 16.5 | 4 | This study |
| *Phrynobatrachus ungujae* | MCZ A-138445 | Tanzania: Ungujae | 10.7 | 4 | This study |
| *Phrynobatrachus ungujae* | MTSN 5866 | Tanzania: Ungujae | 12.1 | 4 | This study |
| *Phrynobatrachus ungujae* | - | Tanzania: Ungujae | 16.0 | 3.5-4 | Pickersgill, 2007 (N=21) |
| *Phrynobatrachus uzungwensis* | - | Tanzania | 25.0 | 2.25 | Grandison and Howell, 1983 (N=10) |
| *Phrynobatrachus uzungwensis* | BMNH 1982.553 | Tanzania | 22.0 | 1.75 | This study |
| *Phrynobatrachus uzungwensis* | BMNH FJM 851 | Tanzania | 16.6 | 1.5 | This study |
| *Phrynobatrachus uzungwensis* | MTSN 5206 | Tanzania | 19.9 | 2.5 | This study |
| *Phrynobatrachus uzungwensis* | MTSN 5275 | Tanzania | 19.9 | 2.5 | This study |
| *Phrynobatrachus uzungwensis* | MTSN 5498 | Tanzania | 22.4 | 2.5 | This study |
| *Phrynobatrachus uzungwensis* | MTSN 5871 | Tanzania | 22.0 | 2.5 | This study |
| *Phrynobatrachus uzungwensis* | MTSN 5872 | Tanzania | 21.3 | 2.5 | This study |
| *Phrynobatrachus uzungwensis*(cf.) | BM 2005.181 | Tanzania | 16.4 | 1.5 | Specimen |
| *Phrynobatrachus versicolor* | CAS-SUA 13008 | Dem. Rep. Congo | 34.2 | - | This study |
| *Phrynobatrachus versicolor* | MCZ A-17532 | Dem. Rep. Congo | 23.9 | 3 | This study |
| *Phrynobatrachus versicolor* | MCZ A-17533 | Dem. Rep. Congo | 21.9 | 3 | This study |
| *Phrynobatrachus versicolor* | MCZ A-25446 | Dem. Rep. Congo | 26.0 | 3 | This study |
| *Phrynobatrachus versicolor* | MCZ A-25447 | Dem. Rep. Congo | 25.1 | 3 | This study |
| *Phrynobatrachus versicolor* | MCZ A-25448 | Dem. Rep. Congo | 30.8 | 3 | This study |
| *Phrynobatrachus versicolor* | MCZ A-25450 | Dem. Rep. Congo | 31.5 | 2 | This study |
| *Phrynobatrachus villiersi* | - | - | 16.0 | 4 | Rödel and Ernst, 2002 (N=285); Perret, 1988 |
| *Phrynobatrachus werneri* | - | - | 23.0 | 4 | Perret, 1966 (N=26) |
| *Phrynobatrachus werneri* | MVZ 234835 | Cameroon | 16.1 | 4 | This study |
| *Phrynobatrachus werneri* | ZMB 20434 | Cameroon | 18.0 | 4 | This study |
| *Poyntonia paludicula* | - | South Africa | 30.0 | 3.5 | Channing and Boycott, 1989 |
| *Ptychadena mascareniensis* | MCZ A-136734 | Cameroon | 34.9 | 3 | This study |
| *Ptychadena mascareniensis* | MCZ A-137027 | Malawi | 40.3 | 2 | This study |
| *Ptychadena oxyrhynchus* | - | - | 85.0 | - | Channing, 2001 |
| *Pyxicephalus adspersus* | - | - | 230.0 | 3 | Channing and Howell, 2006 |
| *Strongylopus faciatus* | - | - | 50.0 | 4 | Channing, 2001 |
| *Strongylopus grayii* | - | - | 64.0 | - | Channing, 2001 |
| *Tomopterna cryptotis* | - | - | 58.0 | - | Channing, 2001 |
| *Tomopterna natalensis* | MCZ A-106532 | Swaziland | 36.0 | 3.5 | This study |
| *Tomopterna natalensis* | MCZ A-20787 | South Africa | 40.7 | 3 | This study |
| *Tomopterna natalensis* | MCZ A-22264 | South Africa | 27.5 | 3 | This study |
| *Tomopterna natalensis* | MCZ A-22265 | South Africa | 27.7 | 3 | This study |
| *Tomopterna natalensis* | MCZ A-22266 | South Africa | 26.8 | 3.5 | This study |
