# Supplementary material for: Terrestrialization, Miniaturization and Rates of Diversification in African Puddle Frogs (Anura: Phrynobatrachidae)
Source: PLoS One. 2012 Apr 10;7(4):e35118. doi: 10.1371/journal.pone.0035118 (PMC3325629; doi:10.1371/journal.pone.0035118)
Supplement: Table S2 — Known reproductive modes of species included in this study. (DOC) [file pone.0035118.s005.doc]

**Table S2. Known reproductive modes of species included in this study.**

| Species | Reproductive Life History | Reproductive Mode | Data Source |
| --- | --- | --- | --- |
| *Amietia angolensis* | aquatic eggs and tadpoles | 0 | Channing, 2001; Channing and Howell, 2006 |
| *Arthroleptella villiersi* | lays its eggs in moss or similar vegetation, which develop directly, without a larval stage | 3 | Channing, 2001; Minter et al., 2004 |
| *Cacosternum boettgeri* | aquatic eggs and tadpoles | 0 | Channing, 2001 |
| *Cardioglossa gracilis* | aquatic eggs and tadpoles | 0 | Amiet, 1972; Perret, 1966 |
| *Conraua crassipes* | aquatic eggs and tadpoles | 0 | Amiet et al., 2004 |
| *Natalobatrachus bonebergi* | clutches (75-100 eggs) deposited above streams; aquatic tadpoles | 1 | Channing, 2001 |
| *Petropedetes martiensseni* | eggs deposited on wet rocks along streams, larvae develop in a thin flim of water as they cling to rocks | 1 | Loader and Howell, 2004; Channing and Howell, 2006; |
| *Petropedetes parkeri* | eggs deposited on large rock surfaces on cliffs and guarded by the male; tadpoles develop terrestrially on cliff surfaces | 1 | Amiet, 1989, 1991, 2004 |
| *Phrynobatrachus acridoides* | aquatic eggs; presumed feeding aquatic tadpoles | 0 | Harper and Vonesh, 2003; Pickersgill 2007; Harper et al. 2010 |
| *Phrynobatrachus alleni* | aquatic eggs and aquatic, feeding tadpoles | 0 | Rödel, 2000 |
| *Phrynobatrachus bullans* | aquatic eggs and presumably aquatic tadpoles | 0 | Crutsinger et al., 2004 |
| *Phrynobatrachus calcaratus* | aquatic eggs (75-220); aquatic, feeding tadpoles | 0 | Barbault and Pilorge, 1980; Rödel, 2000 |
| *Phrynobatrachus dendrobates* | eggs deposited off of the ground, usually in tree holes or above streams; paternal care of eggs observed; aquatic tadpoles (tadpole not yet formally described) | 1 | Drewes et al., 2004a; Drewes, pers. comm. |
| *Phrynobatrachus dispar* | aquatic eggs and tadpoles | 0 | Drewes, pers. comm. |
| *Phrynobatrachus francisci* | aquatic eggs (1000-2500); aquatic, feeding tadpoles | 0 | Lamotte and Dzieduszycka, 1958; Schiøtz, 1964; Lamotte and Xavier, 1966; Rödel, 2000 |
| *Phrynobatrachus graueri* | aquatic eggs and tadpoles (tadpole not yet formally described) | 0 | Drewes et al., 2004 |
| *Phrynobatrachus guineensis* | clutches (30-40 large eggs) deposited above water in small water-filled tree holes empty fruit capsules, or snail shells; feeding, aquatic tadpoles | 1 | Rödel 1998; Rödel and Ernst 2002b |
| *Phrynobatrachus gutturosus* | aquatic eggs and presumably aquatic tadpoles | 0 | Schiøtz, 1964; Rödel, 2000 |
| *Phrynobatrachus krefftii* | clutches (15-30 eggs) deposited on rocks or vegetation above the water; aquatic tadpoles | 1 | Harper and Vonesh, 2003; Channing and Howell, 2006; Pickersgill, 2007; Harper et al. 2010 |
| *Phrynobatrachus latifrons* | aquatic eggs (300-1300); presumably aquatic tadpoles | 0 | Schiøtz, 1967; Rödel, 2000 |
| *Phrynobatrachus leveleve* | aquatic eggs and tadpoles | 0 | Drewes, pers. comm. |
| *Phrynobatrachus mababiensis* | aquatic eggs and tadpoles | 0 | Channing, 2001; de Sá and Channing, 2003; Channing and Howell, 2006; Pickersgill, 2007 |
| *Phrynobatrachus maculiventris* | presumably aquatic eggs; aquatic tadpoles | 0 | Rödel et al., 2009b |
| *Phrynobatrachus minutus* | presumably aquatic eggs; aquatic tadpoles | 0 | Zimkus, personal observation |
| *Phrynobatrachus natalensis* | aquatic eggs; feeding aquatic tadpoles | 0 | Passmore and Carruthers, 1995; Wager, 1986; Lambiris, 1989; DuPreez, 1996; Lamotte and Xavier, 1966; Rödel, 2000; Channing, 2001; Channing and Howell, 2006; Pickersgill, 2007; Harper et al. 2010 |
| *Phrynobatrachus parvulus* | aquatic eggs and tadpoles | 0 | Pickersgill, 2007 |
| *Phrynobatrachus phyllophilus* | terrestrial clutches (20-40 eggs); feeding, aquatic tadpoles in very small puddles | 1 | Rödel and Ernst, 2002b |
| *Phrynobatrachus sandersoni* | clutches laid on vegetation; non-feeding tadpoles (semi-direct development); female attends eggs at night | 2 | Amiet, 1981 |
| *Phrynobatrachus scheffleri* | aquatic eggs and presumably aquatic tadpoles | 0 | Harper et al. 2010 |
| *Phrynobatrachus steindachneri* | presumably aquatic eggs; aquatic tadpoles | 0 | Zimkus, sequence data from tadpole (MCZ A-139119; Genbank FJ769089) |
| *Phrynobatrachus tokba* | terrestrial clutches (2-7 eggs); non-feeding, non-hatching tadpoles (semi-direct development) | 2 | Chabanaud, 1921; Rödel and Ernst, 2002a |
| *Phrynobatrachus ukingensis* | aquatic eggs and presumably aquatic tadpoles | 0 | Harper et al. 2010 |
| *Phrynobatrachus versicolor* | aquatic eggs; feeding aquatic tadpoles (tadpole not yet formally described) | 0 | Drewes and Pickersgill, 2004b |
| *Phrynobatrachus villiersi* | eggs deposited in dried-up puddles prior to rains; larvae develop in very small puddles | 1 | Rödel and Schiøtz, 2004 |
| *Poyntonia paludicola* | aquatic eggs and tadpoles | 0 | Channing, 2001 |
| *Ptychadena mascareniensis* | aquatic eggs and tadpoles | 0 | Channing, 2001; Channing and Howell, 2006 |
| *Pyxicephalis adspersus* | aquatic eggs and tadpoles | 0 | Channing, 2001 |
| *Strongylopus fasciatus* | eggs are laid singly in shallow water on the edges of streams or other bodies of water with grassy margins | 0 | Channing, 2001; Channing and Howell, 2006 |
| *Tomopterna natalensis* | aquatic eggs and tadpoles | 0 | Channing, 2001 |
